# Dynamics and plasticity of the epithelial to mesenchymal transition induced by miR-200 family inhibition

**DOI:** 10.1038/srep21117

**Published:** 2016-02-18

**Authors:** Takeshi Haraguchi, Masayuki Kondo, Ryo Uchikawa, Kazuyoshi Kobayashi, Hiroaki Hiramatsu, Kyousuke Kobayashi, Ung Weng Chit, Takanobu Shimizu, Hideo Iba

**Affiliations:** 1 Division of Host-Parasite Interaction, Department of Microbiology and Immunology, Institute of Medical Science, University of Tokyo Div. Host-Parasite Interaction, Int. Med. Sci., Univ. Tokyo 4-6-1 Shirokanedai, Minato-ku, Tokyo 108-8639, Japan

## Abstract

Whereas miR-200 family is known to be involved in the epithelial-to-mesenchymal transition (EMT), a crucial biological process observed in normal and pathological contexts, it has been largely unclear how far the functional levels of these tiny RNAs alone can propagate the molecular events to accomplish this process within several days. By developing a potent inhibitor of miR-200 family members (TuD-141/200c), the expression of which is strictly regulatable by the Tet (tetracycline)-On system, we found using a human colorectal cell line, HCT116, that several direct gene target mRNAs (*Zeb1/Zeb2, ESRP1, FN1*and *FHOD1*) of miR-200 family were elevated with distinct kinetics. Prompt induction of the transcriptional suppressors, Zeb1/Zeb2 in turn reduced the expression levels of *miR-200c/-141* locus, *EpCAM, ESRP1* and *E-Cad.* The loss of ESRP1 subsequently switched the splicing isoforms of *CD44* and *p120 catenin* mRNAs to mesenchymal type. Importantly, within 9 days after the release from the inhibition of miR-200 family, all of the expression changes in the 14 genes observed in this study returned to their original levels in the epithelial cells. This suggests that the inherent epithelial plasticity is supported by a weak retention of key regulatory gene expression in either the epithelial or mesenchymal states through epigenetic regulation.

The epithelial-to-mesenchymal transition (EMT) is an essential biological process during normal development but is also observed in a pathological context including human cancer^1^,^2^. It is now well established that key regulators of EMT include the microRNA-200 (miR-200) family members which are produced from polycistronic RNAs transcribed from chromosome 12 (miR-200c and -141) and from chromosome 1 (miR-200b, -200a and -429), respectively^3^. The miR-200 family members have been reported to inhibit EMT and enhance the reverse process i.e. the mesenchymal-to-epithelial transition (MET)^3^,^4^,^5^. Important target genes of miR-200 families have been intensively reported^3^,^6^,^7^,^8^,^9^,^10^. And several molecular events induced by the changes in miR-200 activity have been shown to be involved in EMT^11^,^12^,^13^,^14^,^15^,^16^. However, the interrelationship among the events largely remains fragmented.

We have previously developed plasmid- or retro/lentivirus-based expression vectors for a decoy RNA designated as “Tough Decoy (TuD)”^17^, which targets and inhibits certain miRNAs specifically and efficiently. The TuD RNA molecule has a unique secondary structure comprising two miRNA binding sites and has been shown to have very potent miRNA inhibitory functions in comparison with other vector-based miRNA inhibitors. It has thus been widely adopted in several biological systems^18^,^19^. EMT can be induced by miR-200c inhibition in a colorectal tumor cell line, HCT 116, by 5 consecutive transfections of antisense oligonucleotides for this miRNA every 3 days or by a single transduction of a lentivirus vector expressing TuD-200c (TuD designed for inhibiting miR-200c), and subsequent passaging for 11 days^3^,^20^. These observations suggest that more than one week would be required for the establishment of the dramatic overall shifts to the gene regulatory networks in mesenchymal-like cells and also indicate that the transduction of a TuD virus vector would be a more convenient method for the long-term suppression of miRNA activity. However, because of the eclipse periods required for viral entry and integration before TuD RNA transcription can commence, and also because of the drug selection required for generating transductants, detailed kinetic analysis of the molecular processes that occur after the inhibition of specific miRNAs by TuD has not been previously possible. The ability to shut-off TuD expression at certain experimental time points would be a powerful tool to examine whether the suppression of specific miRNA activity in some cell-types would irreversibly establish new regulatory networks.

In our present study, we have developed a tetracycline (Tet)-inducible expression system (Tet-on) for TuD RNA to analyze the EMT induced by the functional suppression of the entire miR-200 family. We thereby examined the molecular events that establish new gene-regulatory networks in mesenchymal like-cells and also tested the plasticity of epithelial phenotypes.

## Results

### Development of a tetracycline-inducible TuD RNA expression system

We first selected the most potent PolIII promoters from mouse *U6*, human *HI*, human *7SK* and a modified form of 7SK (e7SK) (Supplementary Figure S1a), based on the results of previous reports^21^,^22^. Among these promoters, the e7SK promoter was placed upstream of the TuD-21 producing sequence and showed the highest miRNA inhibitory activity and almost canceled out the RNA interference induced by endogenous miR-21 in a luciferase reporter system (Supplementary Figure S1b and Supplementary Figure S2). We thus selected the e7SK promoter as the parental vector for the regulatable constructs. Additionally, when TuD expression plasmids containing this PolIII promoter were transfected into cells, the expression of such interferon response genes as *OAS1, OAS2, MX1, IRF9* and *IFITM1* was undetectable in each case (Supplementary Figure S3), indicating that no unintended immunostimulation was induced by any TuD transcript.

To develop a Tet-inducible PolIII-promoter driven TuD RNA expression system, the optimal sites and number of O2-type tetracycline operator sequences were screened by constructing 10 types of e7SK promoter derivatives (#1–#10) (Supplementary Figure S4a)^23^,^24^. We also selected a stable HCT116 clone harboring the tTR-KRAB expression lentivirus vector (pXL001) for clear switching of TuD expression and designated this transductant as HCT116-TetOnIII. By transfecting expression plasmids for TuD-21 driven by each of 10 e7SK promoter derivatives, we found that the Tet#6 and Tet#10 promoters did not inhibit miR-21 activity at all in the absence of Dox but had full inhibitory effects in the presence of Dox (Supplementary Figure S4b). We designated the #6 promoter as the Tete7SK promoter thereafter and used it for further analysis.

The Tete7SK promoter was placed upstream of the TuD-200c DNA insert and then introduced into a lentivirus vector (Supplementary Figure S5a). When HCT116-TetOnIII cells were transduced with this vector, no miRNA inhibitory effects was observed in the absence of Dox in a reporter assay, whereas almost full suppression of endogenous miR-200c activity was observed at a Dox dosage of 10 ng/ml-1 μg/ml (Supplementary Figure S5b). Since no cytopathic or cytostatic effects are observed over this concentration range of Dox, this system showed its applicability for the analysis of all-or-none switching of endogenous miRNA, and TuD expression was induced at a 0.1 μg/ml Dox level in this system in further experiments.

### EMT and MET induced by the regulated inhibition of miR-200 family activity

**Figure 1:**
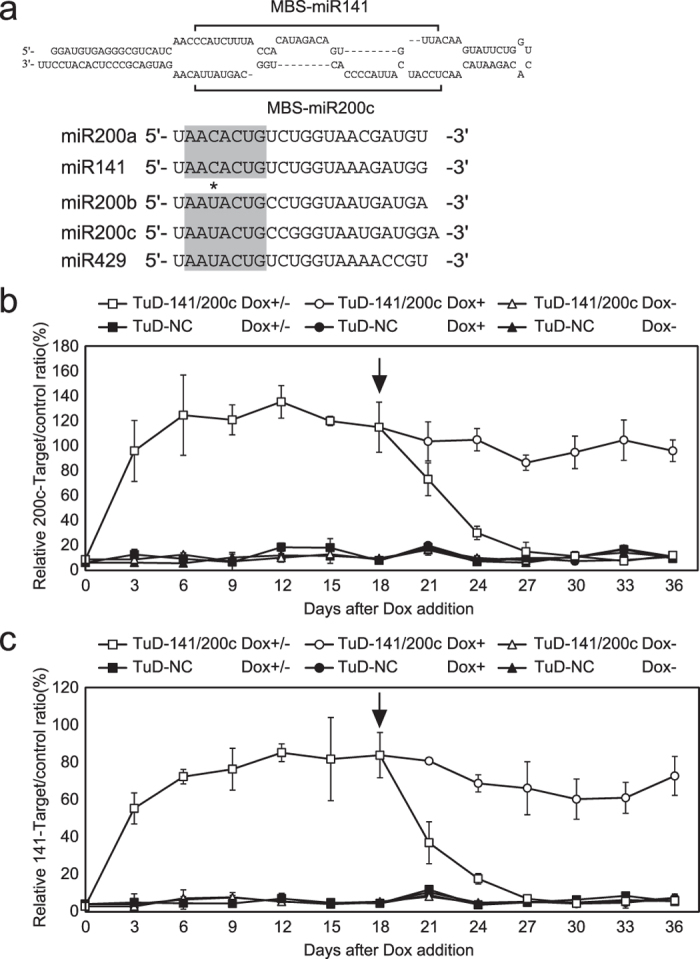
MiR-200c and-141 were inhibited in a Dox-dependent manner using the Tet-inducible TuD-141/200c expression system. (**a**) Sequence and structure of TuD-141/200c, a hybrid TuD RNA which inhibits miR-200 family members simultaneously. Sequences of miR-200 family members as well as their seed sequences (grey box) are shown in the lower panel. The asterisk indicates the nucleotides that differ between miR-200a/141 and miR-200b/c/429. (**b**,**c**) Time course analysis of endogenous miR-200c (**b**) or miR-141 (**c**) activity. HCT116-TetOnIII cells transduced with pLSB-Tete7SK-TuD-141/200c (TuD-141/200c) or pLSB-Tete7SK-TuD-NC (TuD-NC) were cultured either in the absence (Dox-) or presence (Dox+) of Dox. On day 18, Dox was removed from 50% of the cultures which were then further grown (Dox+/−). The cultures were transfected with dual luciferase reporters at 48 hours before the indicated times. The expression ratios of miR-200c-RL/FL to UT-RL/FL or miR-141-RL/FL to UT-RL/FL are represented by the mean ± SD (n = 3). Black arrows indicate the time point of Dox removal.

The cumulative evidence to date indicates that the miR-200 family members are among the key regulators of the EMT. MiRNA microarray analysis of HCT116 cells indicated that two miR-200 loci are transcribed at basal levels, whereas production of miR-200c/-141 (transcribed from chr. 12) is much higher than that of the other miR-200 members (which are transcribed from chr. 1) (Supplementary Figure S6). It was also recently reported that because of a single nucleotide difference in their core sequences (2 to 8 nt from the 5′ end), miR-200a and miR-200b have distinct target specificities, whereas a considerable number of target genes overlap between them^10^. Considering that the core sequence of miR-200c is shared by miR-200b and -429, whereas that of miR-200a is identical to miR-141 (Fig. 1a), we designed a hybrid type TuD molecule with 2 miRNA binding sites (MBS) that are complementary to miR-200c and miR-141, respectively. Since each MBS in TuD can efficiently suppress the activity of miRNAs with the same core sequence^17^, this TuD hybrid, TuD-141/200c, would therefore efficiently suppress almost the entire miR-200 family (Fig. 1a).

We transduced the Tete7SK-TuD-141/200c lentivirus vector in HCT116-TetOnIII cells and after selection obtained the HCT116-TetOn-TuD-141/200c line. These cell cultures were continuously maintained either in the absence of Dox (Dox-) or exposed to Dox on day 0 (Dox+). In half of the Dox+ cultures, Dox was removed on day 18 (Dox+/−) and the other half were maintained in the presence of Dox (Dox+).

We determined the activities of both miR-200c and miR-141 by measuring luciferase activity of the reporter plasmids which were transfected 2 days before the time indicated (Fig. 1b,c, respectively). On day 6, the reporter activity for either miR-200c or miR-141 in Dox+ cells reached similar levels to that of the reporters without the target sequence (untargeted, UT) indicating that RNA interference by either miR-200c or miR-141 had been almost fully suppressed. In contrast, the activities of miR-200c and miR-141 had almost reverted to their original states by day 27 in the Dox+/− cells (9 days after Dox removal). From our reduction kinetic analysis of reporter activity after Dox removal, we were able to roughly estimate the half-life of TuD-141/200c molecule *in vivo* at around 2.2 days.

**Figure 2:**
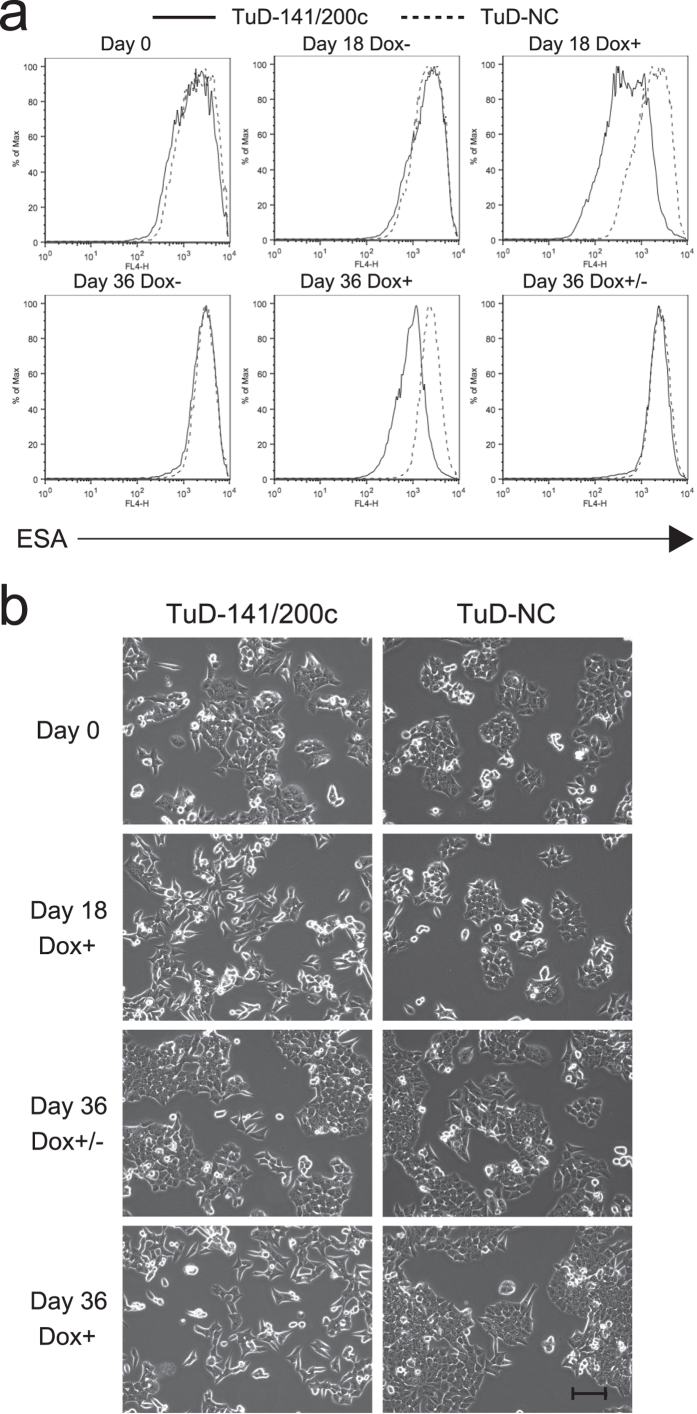
EMT is induced by pLSB-Tete7SK-TuD-141/200c. (**a**) The ESA expression profiles of the parallel cultures described in Fig. 1b were analyzed at the times indicated by FACS. (**b**) The cellular morphologies were then observed under a phase-contrast microscope. Bar, 100μm.

We examined the expression profiles of ESA (epithelial specific antigen, EpCAM) by FACS (Fig. 2a and Supplementary Figure S7) and observed the cellular morphology in these processes every 3 days (Fig. 2b). Dox+ cells began to lose their cuboidal structure and to assume an elongated mesenchymal-like morphology at around day 6. The entire peak of the ESA expression profile of Dox+ cells was shifted to a peak of about 25% of that seen in the Dox- cells at day 9, indicating that EMT had occurred in the entire cell population of these cultures. In the Dox+/− cells, the ESA expression levels detected by FACS had fully returned to a Dox- state by day 30 (12 days after Dox removal) (Supplementary Figure S7). Using similary treated cells harboring TuD-141/200c, we have doubly immunostained cells with anti-E-cadherin (Red) and anti-vimentin (Green) (Supplementary Figure 8). Whereas cells maintained in the absence of Dox expressed E-cadherin but not vimentin, cells maintained in the presence of Dox for 12 days expressed only vimentin. Importantly, when these cells were further maintained in the absence of Dox for 12 days, they were similarly stained as cells continuously maintained in the absence of Dox. Cells harboring TuD-NC expressed only E-Cadherin independent of Dox treatment. Overall these observations support that miR-200-mediated EMT is reversible in this cell line.

### Kinetics of the multiple molecular events involved in the EMT induced by inhibition of the miR-200 family

It has been extensively reported that the transient expression of miR-200 family inhibitors induces a wide-variety of molecular events that support EMT processes^3^,^6^,^7^. However, the long-term kinetics of these pathways have not been fully analyzed previously and knowledge of the interplay between these events has remained fragmented. Using our system described above for regulating miR-200 family activity, we performed time course analysis of the key molecular events that have been previously reported to be involved in the EMT.

**Figure 3:**
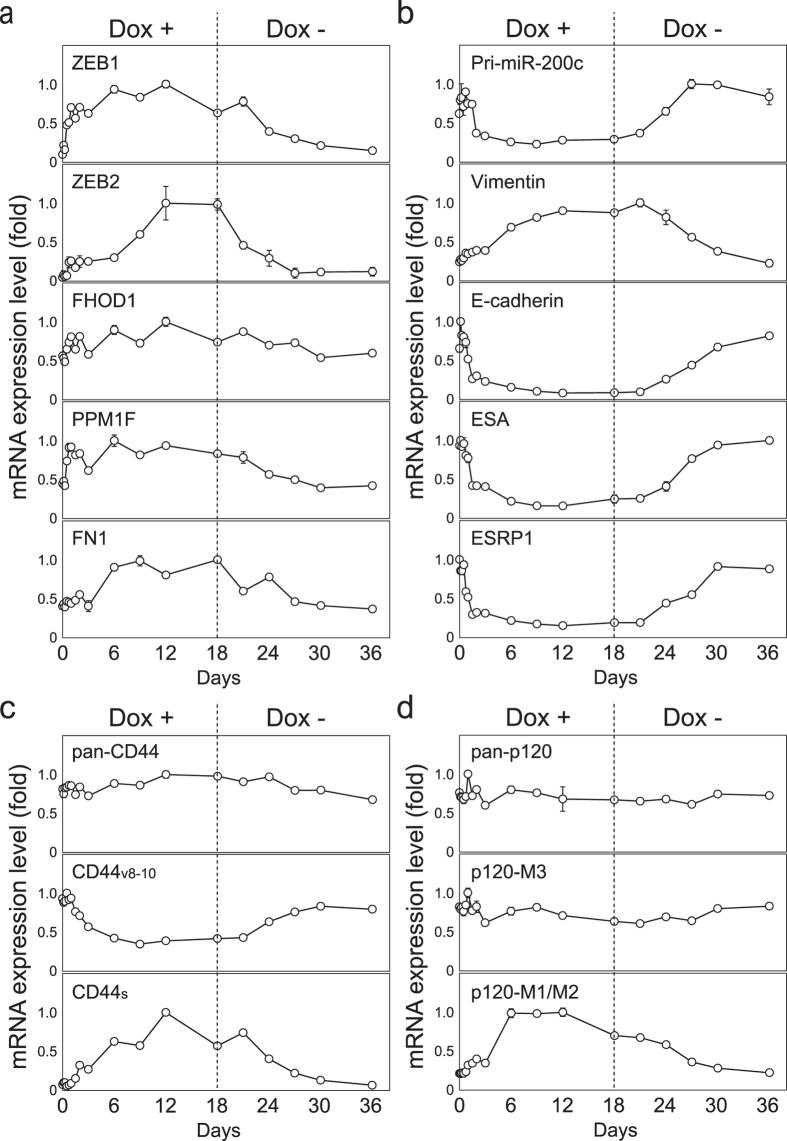
Expression levels of the indicated mRNAs after TuD-141/200c induction and a release from the functional inhibition of the miR-200 family. Total RNA was prepared from Dox+ cultures (days 1–18) and Dox+/− culture (days 18–36) and the mRNA levels were assayed by qRT-PCR. The analyzed genes included (**a**) *Zeb1, Zeb2, FHOD1, PPM1F* and *FN1*; (**b**) *pri-miR200*c/141, *vimentin, E-Cadherin, ESA* and *ESRP1*; (**c**) Total, epithelial type isoform and mesenchymal type isoform of *CD44*; and (**d**) total, epithelial type isoform and mesenchymal type isoform of *p120 catenin*. Dotted lines indicate the time point of Dox removal. For each gene, the expression ratios of the transcripts in HCT116-TetOn-TuD-141/200c cells to those in HCT116-TetOn-TuD-NC cells at the same time points of Dox treatment were calculated and the expression levels are represented by the mean ± SD (taking the maximum level as 1.0) (n = 3).

As early as 12–16 hours after Dox addition, representative miR-200 family targets, *Zeb1* and *Zeb2* began to increase their levels by more than 5-fold, indicating that their mRNAs are extremely sensitive to a reduction in miR-200 activity (Fig. 3a and Supplementary Figure S9). This also reflects the fact that both mRNAs contain multiple strong target sequences for miR-200 family members in their 3′-UTR regions^3^,^6^. Of note, Zeb2 expression reached its highest levels 12 days after TuD-141/200c induction, suggesting some transcription factors triggered by TuD-141/200c might secondarily enhance Zeb2 transcription. Other miR-200 family targets, including *FHOD1, PPMIF and FN1* mRNAs, were induced in a lesser extent with distinct kinetics for each gene (Fig. 3a). For example, *FN1* expression was not significantly elevated for the first 3 days and reached its maximum level on day 6 (Fig. 3a and Supplementary Figure S9), probably reflecting a less effective miR-200c binding site in *FN1* mRNA 3′-UTR^7^.

The Zeb1 and Zeb2 transcription factors have been reported to directly suppress genes such as *E-cadherin, ESRP1*, and *ESA* (*EpCAM*)^14^,^15^,^16^. Consistent with this, these transcripts began to show reduced expression by 18–24 hours after the Dox addition (Fig. 3b). On the other hand, a representative mesenchymal marker gene, *vimentin* was induced by Dox in a delayed fashion, probably reflecting the previous observation that it is positively regulated by Zeb1 and Zeb2, probably in an indirect fashion^12^,^13^. Importantly, transcription of the miR-200c/141 locus, which is also known to be under direct negative regulation of Zeb1 and Zeb2^11^, was also suppressed in the Dox+ cells as judged by the reduction in the pri-miR-200c/-141 transcript level using a primer pair that does not detect mature miR-200c or miR-141. This would lead to the down-regulation of endogenous miR-200c/-141 production, confirming the establishment of a double-negative feedback loop between Zeb1/2 and the miR-200 family members in a quite early stage of EMT. Interestingly, the down-regulation of *ESRP1* mRNA, whose product is a representative splicing factor for epithelial-specific isoform production, was found to be associated with partial isoform switching exemplified by the increased levels of mesenchymal mRNA isoforms of CD44 (CD44s (standard)) or p120 catenin (p120 M1/M2)^25^, respectively (Fig. 3c,d). Concomitantly, expression of the corresponding epithelial mRNA isoforms, CD44v8-10 and p120 M3, was reduced, whereas the mRNA levels for all the isoforms detected by PCR did not change significantly.

**Figure 4:**
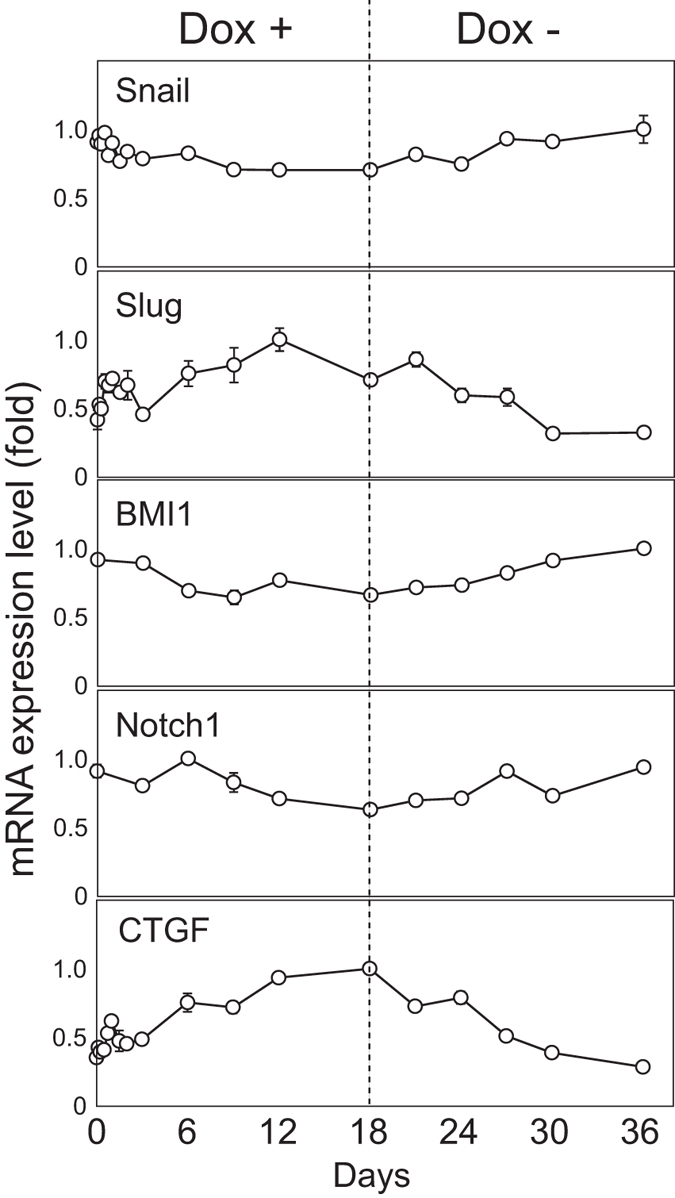
Expression levels of specific genes after TuD-141/200c induction and a release from the functional inhibition of the miR-200 family. The mRNA levels were assayed as described in Fig. 3. The analyzed genes included Snail, Slug, BMI1, Notch1 and CTGF. Dotted lines indicate the time point of Dox removal. For each gene, expression ratios of the transcripts in HCT116-TetOn-TuD-141/200c cells to those in HCT116-TetOn-TuD-NC cells at the same time points of Dox treatment were calculated and the expression levels are represented by the mean ± SD (taking the maximum level as 1.0) (n = 3).

We also analyzed the transcript levels of *Snail, Slug, BMI1, Notch1* and *CTGF*, which are known to be involved in EMT in several cellular systems. Whereas *Slug, BMI1* and *Notch1* have also been reported to be miR-200 family target genes^8^,^9^,^10^, only *Slug* responded to the Dox treatment, and only slightly (Fig. 4). The mRNA encoding Twist, a well-known transcription factor involved in EMT process, was undetectable by RT-PCR in this cell line. Of note, *CTGF* mRNA was induced by the miR-200 inhibition and repressed by the release from this inhibition with a slightly delayed kinetics compared with those of *vimentin*. An miR-200 target, FHOD1 (formin homologue domain-containing protein 1) (Fig. 3a), as a member of the formins, is expected to contribute to actin polymerization (F-actin formation) during EMT. Since the resultant G-actin reduction would release a G-actin binding protein MKL1 from cytoplasmic retention, it is possible that a representative MKL-1 dependent SRF target gene, CTGF is partly induced by this sequential pathway, including actin-remodeling. The elucidation of these entire pathways will be important in the future.

**Figure 5:**
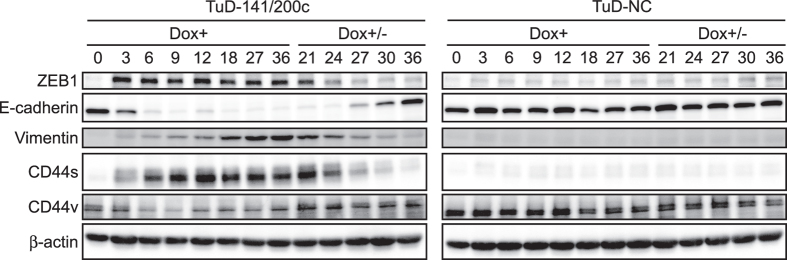
Protein expression levels of the indicated genes after a release from the functional inhibition of the miR-200 family. Total protein was prepared from Dox+ (days 1–18) and Dox+/− (days 18–36) cultures and protein levels were determined by western blotting using actin as an internal control. The analyzed genes included Zeb1, E-cadherin, vimentin, CD44s and CD44v.

When the expression levels of Zeb1, E-cadherin, and vimentin were analyzed by western blotting, a clear induction and subsequent stable expression of Zeb1, as well as a drastic reduction of E-cadherin after Dox treatment, were confirmed (Fig. 5). Accumulation of vimentin protein was quite delayed in comparison with its mRNA levels, probably reflecting a long half-life of the protein. During EMT, a clear induction of CD44s and slight reduction of CD44v protein were also confirmed by western blotting. Overall, these findings indicated that the inhibition of miR-200 family activity alone can trigger several layers of molecular events in a sequential manner including RNA interference, transcriptional repression and activation, switching of splicing isoforms and probably actin remodeling and subsequent SRF target gene induction.

### Reversion of the observed multiple molecular events after the release from miR-200-family inhibition

Upon removal of Dox on day 18, the Dox+/− cells gradually returned to an epithelial expression signature when analyzing all the parameters examined in the EMT process i.e. direct miR-200 targets (*Zeb1, Zeb2, FHOD1, PPMIF* and *FN-1*mRNA), Zeb1/Zeb2 regulated genes (*miR-200c/141* locus, *ESA, ESRP1, E-cadherin* and *Vimentin*), splicing isoforms of *CD44* and *p120 catenin* and the SRF target gene, *CTGF* (Figs 3 and 4). Their mRNA expression levels in the Dox+/− cells were very similar at day 0 and day 36. Protein analysis of Zeb1, E-cadherin, vimentin and CD44s also showed that their expression levels in Dox+/− cells on day 36 were very similar to those in these cells on day 0. This finding indicates that the transcription of the key regulatory genes examined here including Zeb1 and Zeb2 is not strongly retained in either the epithelial or mesenchymal states due to epigenetic regulation under our experimental conditions. This suggests that the corresponding promoters can easily switch from a poised to active state^26^. The observed cascade of molecular events induced by modulation of the functional miR-200 family activity is schematically represented in Supplementary Figure S10.

### EMT and MET are also regulated by the activities of miR-200 family members in the SUM149PT triple-negative breast cancer cell line

**Figure 6:**
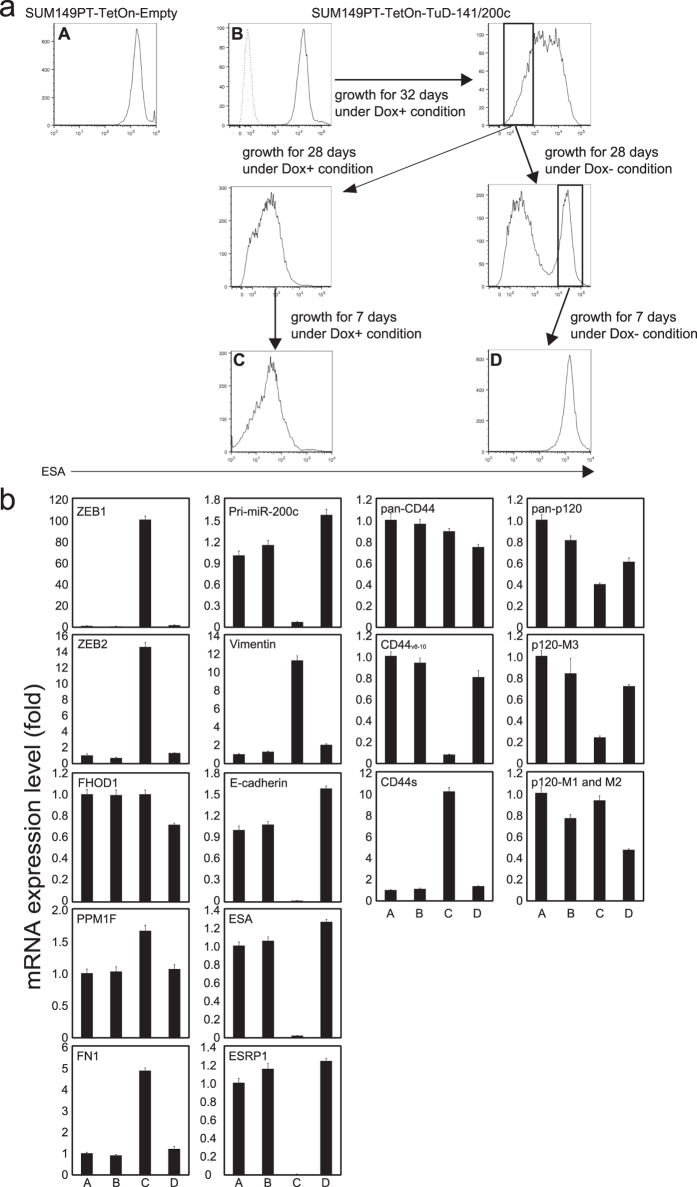
Characterization of a mammary tumor cell line, “SUM149PT-TetOn-TuD-141/200c” which harbours the Tet-inducible TuD-141/200c expression system. (**a**) Schema for the treatment and ESA expression pattern of SUM149PT-TetOn-TuD-141/200c cells. SUM149PT cells were transduced with pXL001 and subsequently with either pLSB-Empty or pLSB-Tete7SK-TuD-141/200c and selected (cultures A,B). The pLSB-Empty transduced cells were cultured in the absence of Dox. Culture B was grown in the presence of Dox for 32 days and the cellular fraction with very low ESA levels was then sorted. One part of this fraction was subsequently cultured for 35 days in the presence of Dox (culture C), whereas the other part was cultured in the absence of Dox for 28 days from which the ESA(+) fraction was sorted and further grown in the absence of Dox for 7 days (culture D). (**b**) Expression levels of several mRNAs after TuD-141/200c induction and a release from its inhibiting activity. Total RNA was prepared from cultures (A–D) described in **(a)**, and all of the mRNAs examined in Fig. 3a–d were determined by qRT-PCR. The relative expression levels are represented by the mean ± SD, taking the level of culture A as 1.0 (n = 3).

To test whether our observations that the EMT induced by miR-200 suppression is highly reversible could be extended to another cell line, we tested the mammary tumor cell line, SUM149PT, as it has been reported to show phenotypic equilibrium in monolayer culture^27^. We transduced SUM149PT cells with pXL001 and subsequently with pLSB-Tete7SK-TuD-141/200c basically as described for HCT116. However, we did not clone SUM149PT cells after pXL001 introduction due to the phenotypic heterogeneity of SUM149PT cultures, whereas HCT116 cells were cloned after pXL001 transduction to select for cells that showed clear switching of TuD expression. When the transduced SUM149PT cells were treated with Dox, we found a more drastic reduction of ESA expression than in HCT116 cells, although full transition was longer in the SUM149PT cells (Fig. 6a). This slower transition might have been partly due to the lack of a cloning process after pXL001 introduction. After 32 days of Dox treatment, we sorted a cellular fraction of the SUM149PT culture with very low ESA levels by FACS and maintained these cells either in Dox+ or in Dox- conditions for an additional 28 days. A cellular fraction expressing ESA at high levels was detected only in the Dox- culture. Thus, 28 days appeared to be insufficient for a full transition, probably because of slow transition rate in SUM149PT cells and also because of a leaky expression of TuD-141/200c in the uncloned cell populations. However, we could not exclude the possibility that ESA(−) cells had retained their properties through some epigenetic mechanisms.

RNA was prepared from cells in cultures A–D shown in Fig. 6a and the expression levels of all genes examined for HCT116 shown in Fig. 3a–d were analyzed (Fig. 6b). Expression changes during Dox addition and removal were very similar to those in the HCT116 system; culture B (original ESA(+)) and culture D (ESA(+) cells that were derived from ESA (−) cells) had very similar expression levels of all of the examined mRNAs. These patterns of expression were also very close to those in culture A, which was transduced with pXL001 and pLSB-empty and maintained in the absence of Dox. Importantly in culture C (ESA(−) cells originated from ESA(+) cells after the TuD-141/200c induction), Zeb1 and Zeb2 were drastically elevated among the miR-200 family targets. Whereas we observed Zeb1 and Zeb2 activation in the HCT116 cell system, this was much stronger in the SUM149PT cultures. As a result, pri-miR-200c, E-cadherin, ESA and ESRP1 were more efficiently suppressed in SUM149PT culture C due to the highly elevated Zeb1/2. The striking reduction of ESRP1 in culture C in turn induced more clear changes in the splicing patterns in CD44 as well as p120 when compared with the HCT116 cell system. Hence, the major cascade of molecular events associated with miR-200-dependent EMT that we observed in HCT116 cell system can be basically recapitulated in SUM149PT cells.

## Discussion

We have developed a valuable tetracycline-inducible expression system (a Tet On system) for TuD RNA that shows a marginal basal level of TuD expression in the absence of Dox and very strong expression of TuD in the presence of Dox which can fully inhibit its target miRNA activity (Fig. 1b,c). The hybrid TuD molecule we have designed and described herein, TuD-141/200c, can very efficiently and simultaneously inhibit miR-141 and miR-200c (Fig. 1b,c). Our preliminary analysis using the inducible TuD-200c lentivirus vector in several cell lines showed reduced EMT induction ability, whereas the activation of broader suite of target genes by our hybrid type TuD seems to have markedly increased the accuracy of our molecular analysis of EMT. Of note, such dual-targeting TuDs were reported to be functional using miR-143/145, miR-146a/203 and miR-16/21^28^, indicating that hybrid type TuD can be broadly used.

Our novel TuD expression system enabled us to sequentially follow the propagation of each molecular event that has been fragmentally reported to be involved in EMT. Importantly, the down-regulation of endogenous miR-200c/141 loci at the early stages of this transition would have reinforced the effects of hybrid TuD-141/200c molecule. When we compared the kinetics of Zeb1 and Zeb2 induction, the period required for reaching saturation levels was significantly delayed in the case of Zeb2, which might suggest some additional regulatory layers are required for its maximum expression.

Our current results indicate that EMT induced by TuD-mediated miR-200 family inhibition is fairly reversible after a release from this inhibition. This is mechanistically supported by the transcriptional reversibility of key genes downstream of the miR-200 family examined here including *Zeb1, Zeb2, FHOD1, PPM1F, E-Cad, ESRP1, ESA*, and *CTGF*. At least within the periods we examined in our present analyses, these genes do not significantly retain their previous expression levels epigenetically. In other words, all of the promoters of these genes can readily switch from a poised to active or from an active to poised state, resulting in the prompt response of these genes in the HCT116 tumor cell line. As expected, isoform switching induced by ESRP1 was found to be completely dependent upon the ESRP1 expression level. Our current results highlight the molecular basis of epithelial plasticity supported by the miR-200 family.

The regulatable TuD expression system was also found to be applicable to another cancer cell line, SUM149PT, which originated from a triple-negative mammary tumor. Interestingly, it has been reported that the SUM149PT line comprises a mixture of cellular populations in phenotypic equilibrium in monolayer culture. Because of this unique property, we did not perform cellular cloning in this line after pXL001 transduction for clear switching of TuD expression. Even with this limitation, we were still able to detect the reversibility of miR-200-mediated EMT in this cell line. Importantly, all of the major mRNAs shown to be involved in miR-200-mediated EMT in HCT116 cells had similar changes in SUM149PT cells upon induction of and release from TuD-141/200c expression, indicating that cascade of molecular events represented in Supplementary Figure S10 can be induced by modulation of the functional miR-200 family activity in different cell types.

The regulatable TuD expression system developed here is thus applicable to different *in vivo* systems. For example, it may be used to test whether suppression of a specific miRNA has therapeutic potential in animal disease models. By screening the periods of Dox treatment, we could also in the future use our system to evaluate the critical timing required for the inhibition of a crucial miRNA and also the required duration of this inhibition. When proofs of concept of this nature are obtained using our TuD expression vector in animal disease models, therapeutic strategies in terms of the dosing periods and dosage amounts required for miRNA inhibitors such as S-TuD will become easier to design and optimize^20^.

## Methods

### Cell culture

The human colorectal adenocarcinoma cell line, HCT116 was obtained from ATCC and was cultured at 37 °C in DMEM containing 10% foetal bovine serum (FBS). For tetracycline induction, cells were cultured at 37 °C in DMEM containing 10% Tet-approved FBS (TaKaRa, Japan) with or without Dox (doxycycline; Sigma-Aldrich, USA). The triple-negative breast cancer cell line, SUM149PT, was obtained from Asterand (USA) and cultured at 37 °C in Ham’s F-12 containing 5% FBS, 10 mM HEPES, 5 μg/ml insulin, 1 μg/ml hydrocortisone and 5 μg/ml gentamicin (SUM149PT medium). In the tetracycline induction experiments, cells were cultured at 37 °C in SUM149PT medium containing 5% Tet-approved FBS with or without 1 μg/ml doxycycline.

### Plasmid construction

For the construction of the H1 promoter-type, e7SK (enhanced 7SK) promoter-type and Tete7SK (Tetracycline-responsive e7SK) promoter type TuD shuttle vectors, The DNA fragments listed in Supplementary Table S1 were synthesized by Genscript (USA). These PolIII-TuD shuttle fragments were digested with BamHI and EcoRI and cloned into the BamHI-EcoRI site of pCR2.1 (Thermo Fisher Scientific, USA) to generate the pH1-TuD-, pe7SK-TuD- and pTete7SK-TuD-shuttle vector, respectively. Two other PolIII-TuD shuttle vectors, pmU6-TuD-shuttle and ph7SK-TuD-shuttle, have been described previously^17^,^20^. For the construction of PolIII promoter driven TuD RNA expression plasmids, a series of oligonucleotide pairs were synthesized (Supplementary Table S2). Each oligonucleotide pair was annealed and cloned into the PolIII-TuD-shuttle vectors digested with BsmBI to generate PolIII-TuD RNA-cassettes. These cassettes were then subcloned into the BamHI-EcoRI site of pSL1180 to generate polIII-driven TuD RNA expression plasmids.

For lentivirus construction, the pLenti6/V5-GW/lacZ (Thermo Fisher Scientific, USA) plasmid was digested with AgeI, treated with Klenow fragment and then digested with KpnI. pLSP was digested with ClaI, treated with Klenow fragment and then digested with KpnI^17^. The resulting 0.9 kb and 5.1 kb fragments were ligated to generate pLSB. To construct the Tet-inducible TuD RNA expression lentivirus vectors, BamHI-EcoRI fragments of pTete7SK-TuD-200c, pTete7SK-TuD-141/200c and pTete7SK-TuD-NC were subcloned into the pLSB lentivirus vector to generate pLSB-Tete7SK-TuD-200c, pLSB-Tete7SK-TuD-141/200c and pLSB-Tete7SK-TuD-NC, respectively. To construct the luciferase reporter plasmids, the oligonucleotide pairs listed in Supplementary Table S3 were annealed and cloned into the XhoI-NotI sites of psiCHECK2 (Promega, USA) to generate psiCHECK2-T21, psiCHECK2-T200c and psiCHECK2-T141, respectively.

### Antibody staining and FACS analysis

Cells were stained with αESA-APC (324208, BioLegend, USA) and analysed by FACS Calibur (BD, USA) or FACS Aria (BD).

### Construction of tetracycline-inducible cell lines

HCT116 cells were seeded at 10^5^ cells per well in six-well plates and transduced with pXL001 (26122^29^, Addgene, USA) viral stock (<1 × 10^4^ TU) in the presence of 8 μg/ml of polybrene and selected with puromycin (1 μg/ml) at 24 hours after transduction. After 10 days of selection, puromycin was removed from the medium. Several stable clones were isolated by FACS sorting using FACS Aria, one of which was chosen and designated as the HCT116-TetONIII stable line. HCT116-TetOnIII cells were seeded at 10^5^ cells per well in six-well plates in DMEM containing 10% FBS. After 24 hours, these cells were transduced with each pLSB-Tete7SK-TuD-141/200c or pLSB-Tete7SK-TuD-NC virus stock (3 × 10^5^ TU) in the presence of 8 μg/ml of polybrene to generate HCT116-TetOn-TuD-141/200c cells and HCT116-TetOn-TuD-NC cells. The medium was then substituted with DMEM containing 10% FBS and blasticidin (10 ug/ml) after a further 24 hours. After seven days of selection, the blasticidin was removed from the medium.

SUM149PT cells were seeded at 10^5^ cells per well in six-well plates and transduced with pXL001 viral stock (<1 × 10^4^ TU) in the presence of 8 μg/ml of polybrene and selected with puromycin (1 μg/ml) at 24 hours after transduction. After 10 days of selection, puromycin was removed from the medium and stable cell populations were designated as SUM149PT-TetONIII cells. SUM149PT-TetONIII cells were then seeded at 10^5^ cells per well in six-well plates. After 24 hours, these cells were transduced with each pLSB-Tete7SK-TuD-141/200c or pLSB virus stock (3 × 10^5^ TU) in the presence of 8 μg/ml of polybrene to generate SUM149PT-TetOn-TuD-141/200c cells and SUM149PT-TetOn-Empty cells. The medium was then substituted with SUM149PT medium containing blasticidin (10 ug/ml) after a further 24 hours. After seven days of selection, the blasticidin was removed from the medium.

### Transfection and Luciferase assays

Cells were seeded at 10^5^ cells per well in 24-well plates in DMEM containing 10% FBS at one day prior to transfection. HCT116-TetOnIII cells were transfected in triplicate with PEI-MAX (Polysciences, USA), 200 ng of dual luciferase target reporter plasmid (Supplementary Figure S2) and 1–30 ng of TuD RNA expression plasmid. HCT116-TetOn-TuD141/200c cells and HCT116-TetOn-TuDNC cells were transfected in triplicate with PEI-MAX and 200 ng of dual luciferase target reporter plasmid. We performed all dual luciferase reporter assays at 48 hours after transfection using the Glomax microplate luminometer (Promega).

### RNA preparation and quantitative RT-PCR

Total RNA was prepared from HCT116-TetOn-TuD141/200c and HCT116-TetOn-TuDNC cells using miRVana (Thermo Fisher Scientific). Total RNA was prepared from SUM149PT-TetOn-TuD-141/200c cells and SUM149PT-TetOn-Empty cells using Directsol (Zymo Research, USA). First strand cDNA was then synthesized using a PrimeScript RT reagent Kit with gDNA Eraser (Takara). Real-time PCR was performed using the StepOne real-time PCR system (Thermo Fisher Scientific) with SYBR Select Master Mix (Thermo Fisher Scientific) as a reporter. The data were normalized using GAPDH expression levels. The sequences of the real-time PCR primers are listed in Supplementary Table S4.

### Western blotting

Total proteins were extracted from cells using 1.5 × SDS denaturing buffer. The protein extracts were separated by 10% SDS-PAGE and transferred onto a PVDF membrane (Millipore, USA). Immunoblotting was performed by incubating the membrane in Can Get Signal Solution I (TOYOBO, Japan) containing antibodies against E-cadherin (#5296, Cell Signaling Technology, USA), vimentin (sc-6260, Santa Cruz, USA), ZEB1 (#3396, Cell Signaling Technology), CD44 (#3570, Cell Signaling Technology), CD44v9 (CAC-LKG-M003, CosmoBio, Japan) and actin (sc-47778, Santa Cruz) for overnight at 4 °C. The membranes were incubated in Can Get Signal Solution II (TOYOBO) containing secondary antibodies conjugated with horseradish peroxidase for 1 hour at RT after three washes with TBS containing 0.1% Tween 20. Signals were detected on an AE-9300H-CP Ez-CaptureMG (ATTO, Japan) imaging analyser using ECL Western Blotting Substrate (Promega) or Immunostar DL (WAKO, Japan).

## Additional Information

**How to cite this article**: Haraguchi, T. *et al*. Dynamics and plasticity of the epithelial to mesenchymal transition induced by miR-200 family inhibition. *Sci. Rep.* **6**, 21117; doi: 10.1038/srep21117 (2016).

## Supplementary Material

Supplementary Information
