# Supplementary material for: Dynamics and plasticity of the epithelial to mesenchymal transition induced by miR-200 family inhibition
Source: Sci Rep. 2016 Feb 18;6:21117. doi: 10.1038/srep21117 (PMC4758077; doi:10.1038/srep21117)
Supplement: Supplementary Information [file srep21117-s1.pdf]

Supplementary Information

Dynamics and plasticity of the epithelial to mesenchymal transition induced by miR-200 family inhibition

Takeshi Haraguchi, Masayuki Kondo, Ryo Uchikawa, Kazuyoshi Kobayashi, Hiroaki Hiramatsu, Kyousuke Kobayashi, Ung Weng Chit, Takanobu Shimizu & Hideo Iba*

Division of Host-Parasite Interaction, Department of Microbiology and Immunology, Institute of Medical Science, University of Tokyo

Div. Host-Parasite Interaction, Int. Med. Sci., Univ. Tokyo 4-6-1 Shirokanedai, Minato-ku, Tokyo 108-8639, JAPAN

*To whom correspondence should be addressed. Email: iba@ims.u-tokyo.ac.jp

Methods

Construction of prototype Tet-inducible TuD expression plasmids.

The synthetic DNA fragments listed in Supplementary Table S5 (synthesized by Genscript) were used for the construction of the Tete7SK #1-#2 promoter type TuD shuttle vectors. These Tete7SK1-TuD and Tete7SK2-TuD shuttle fragments were digested with BamHI and EcoRI and cloned into the BamHI-EcoRI site of pCR2.1 to generate Tete7SK#1-TuD-shuttle and Tete7SK#2-TuD-shuttle vectors, respectively. For the construction of TuD RNA expression plasmids driven by PolIII promoters, a oligonucleotide pair for TuD-21 (Supplementary Table S2) was annealed and cloned into each PolIII-type-TuD-shuttle vector digested with BsmBI to generate the corresponding PolIII-driven TuD-21 expression cassettes. These cassettes were then subcloned into the BamHI-EcoRI site of pSL1180 to generate polIII-driven TuD-21 expression plasmids. For the construction of the Tete7SK #3-#10 promoter-driven TuD-21 vectors, the DNA fragments listed in Supplementary Table S6 (synthesized by Genscript) were used. These Tete7SK #3-#10 promoter type TuD-21 fragments were digested with BamHI and EcoRI and cloned into the BamHI-EcoRI site of pSL1180 to generate Tete7SK #3-#10 promoter-driven TuD RNA expression plasmids.

Immunofluorostaining

Cells were seeded at $5x10^5$ cells per well in eight-well chamber slides, cultured for 2 days, and then fixed in 4% paraformaldehyde PBS (Nacalai Tesque, Japan) and permeabilized with 0.2% Triton X-100 PBS. Blocking was performed using a 1:1 mixture of 5% BSA, 0.02% NaN3 PBS and Blocking one (Nacalai Tesque). Cells were stained by incubating in blocking buffer containing antibodies against E-cadherin (24E10, Cell Signaling Technology, 1:100) and vimentin (sc-6260, Santa Cruz, 1:100) for overnight at 4°*C*. Cells were incubated in blocking buffer containing Alexa Fluor 546 and 488 conjugated secondary antibodies (Thermo Fisher Scientific) for 1hr at 4°*C*. The slides were mounted in Vectashield Mounting Medium with DAPI (Vector Laboratories, USA). Fluorescent images were obtained using a Nikon confocal A1 camera (Nikon, Japan).

Supplementary Figure legends

Supplementary Figure S1. Comparison of polIII promoters for TuD RNA expression. (**a**) Sequences of the h7SK and e7SK promoters. Positions of TATA box, octamer motif, PSE and CACCC box are shown. The modified sequences in the e7SK promoter are highlighted in red. The two arrows in the e7SK promoter indicate the inverted repeat of the octamer motif, which was generated by the modification. (**b**) The miRNA inhibitory activity of TuD RNA expression vectors driven by the polIII promoter. HCT116 cells were transfected with luciferase reporter vector and TuD RNA expression vectors at several doses. A dual luciferase assay was performed 48 hours after transfection. The expression ratios of miR-21-RL/FL to UT-RL/FL are represented by the mean ± SD (n = 3).

Supplementary Figure S2. Structures of the dual luciferase reporter plasmids, psiCHECK2-UT (**a**), psiCHECK2-T21 (**b**), psiCHECK2-T200c (**c**), and psiCHECK2-T141 (**d**) used in this study. The psiCHECK2-T21, -T200c and -T141 constructs have an insertion sequence just downstream of the *Renilla* luciferase gene, which is fully complementary to mature miR-21 (22bp), miR-200c (23bp) and miR-141 (23bp), respectively.

Supplementary Figure S3. Expression levels of interferon response genes in HCT116 cells transfected with TuD RNA expression plasmids driven by polII or polIII. Sixteen hours after transfection, the expression levels of the exogenous TuD were measured by qRT-PCR and represented by the mean ± SD (n = 3). Poly I:C (100ng/ml) was used as a positive control.

Supplementary Figure S4. Tet-inducible polIII promoters constructed and tested in this study. (**a**) Schematic representation of the parental e7SK promoter and its derivatives harboring O2-type tetracycline operator inserts. Arrows indicate the transcription start sites. (**b**) HCT116-TetOnIII cells were cotransfected with a *luciferase* reporter and one of the TuD RNA expression vectors driven by the promoters shown in (**a**) and cultured with or without doxycycline. A dual luciferase assay was performed at 48 hours after transfection. The ratio of miR-21-RL/FL to UT-RL/FL expression levels are represented by the mean ± SD (n = 3).

Supplementary Figure S5. The Tet-inducible TuD RNA expression system. (**a**) Provirus structure of the Tet-inducible TuD RNA expression lentivirus vector, pLSB-Tete7SK-TuD. (**b**) Doxycycline dose-

dependency of the miR-200c inhibitory activity of pLSB-Tete7SK-TuD-200c. HCT116-TetON cells were transduced with pLSB-Tete7SK-TuD-200c or pLSB-Tete7SK-TuD-NC (negative control) and selected under blasticidin. These cells were then transfected with dual luciferase reporter vectors, T200c and UT grown in the presence of Dox at several doses. A dual luciferase assay was performed 48 hours after transfection. The expression ratios of miR-200c-RL/FL to UT-RL/FL are represented by the mean $\pm$ SD (n = 3).

Supplementary Figure S6. Expression levels of miR-141/200c/200b/200a/429 family in HCT116 cells. The miRNA expression levels were determined by miRNA-microarray (Agilent) and are represented by the mean $\pm$ SD (n = 3).

Supplementary Figure S7. FACS analysis of the ESA expression profiles in HCT116-TetOn-TuD-141/200c and HCT116-TetOn-TuD-NC cells. Dox-, Dox+ Dox+/- cultures were prepared as described in Figure 1b. The blue and red lines represent the ESA expression profiles of HCT-116-TetOn-TuD-141/200c and HCT-116-TetOn-TuD-NC cells, respectively.

Supplementary Figure S8. Immunofluorescent staining of E-cadherin and vimentin in HCT116 cells before and after TuD-141/200c induction. HCT116-TetOnIII cells transduced with pLSB-Tete7SK-TuD-141/200c (TuD-141/200c) or pLSB-Tete7SK-TuD-NC (TuD-NC) were cultured in the absence (Dox-) or presence (Dox+) of Dox. On day 13, Dox was removed from 50% of the cultures which were then further grown (Dox+/-). E-cadherin and vimentin were immunostained with specific antibodies and observed under a confocal microscope. Blue; DAPI, Red; E-cadherin, Green; vimentin. Bar, 10μm.

Supplementary Figure S9. Enlarged graphs from Figure 3a further detailing the time course changes in gene expression within 48 hours of TuD-141/200c induction.

Supplementary Figure S10. Schematic representation of the molecular events that are directly or indirectly regulated by the miR-200 family members. The genes in the yellow boxes are direct targets of the miR-200 family. Blue lines indicate genes whose expression levels are regulated by Zeb1 and Zeb2 proteins either negatively or positively at the transcriptional level.

Supplementary Figure S11. Full-length images of the western blots in this study. Arrowheads indicate the position of protein markers (#161-0374; Bio-Rad). β-actin was used as an internal control. Red boxes indicate the cropped images shown in Figure 5. Blots in a black line box originated from the same gel. In the gels indicated by the blue broken line boxes, the same set of protein samples was charged.

Supplementary Figure S1

a

TATA box　Octamer motif　PSE　CACCC box

h7SK promoter

```
-243  CTGCAGTATTTAGCATGCCCCACCCATCTGCAAGGCATTCTGGATAGTGT
-193  CAAAACAGCCGGAAATCAAGTCCGTTTATCTCAAACTTTAGCATTTTGGG
-143  AATAAATGATATTTGCTATGCTGGTTAAATTAGATTTTAGTTAAATTTCC
 -93  TGCTGAAGCTCTAGTACGATAAGCAACTTGACCTAAGTGTAAAGTTGAGA
 -43  TTTCCTTCAGGTTTATATAGCTTGTGCGCCGCCTGGGTACCTC
```

e7SK promoter

```
-244  CTGCAGTATTTGCATATGCAAATAAGGTGGTGGATCGATTCTGGATAGTG
-194  TCAAAACAGCCGGAAATCAAGTCCGTTTATCTCAAACATTTGCATTTTGG
-144  GAATAAATGATATTTGCATTGCTGGTTAAATTAGATTTTAGTTAAATTTC
 -94  CTGCTGAAGCTCTAGTACGATAAGCAACTTGACCTAAGTGTAAAGTTGAG
 -44  ATTTCCTTCAGGTTTATATAGCTTGTGCGCCGCCTGGGTACCTC
```

b

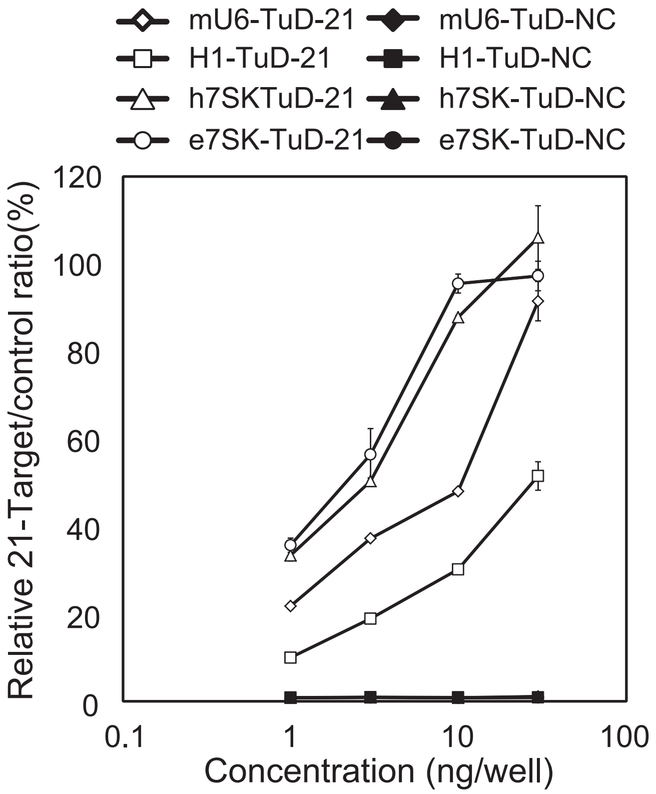

Supplementary Figure S2

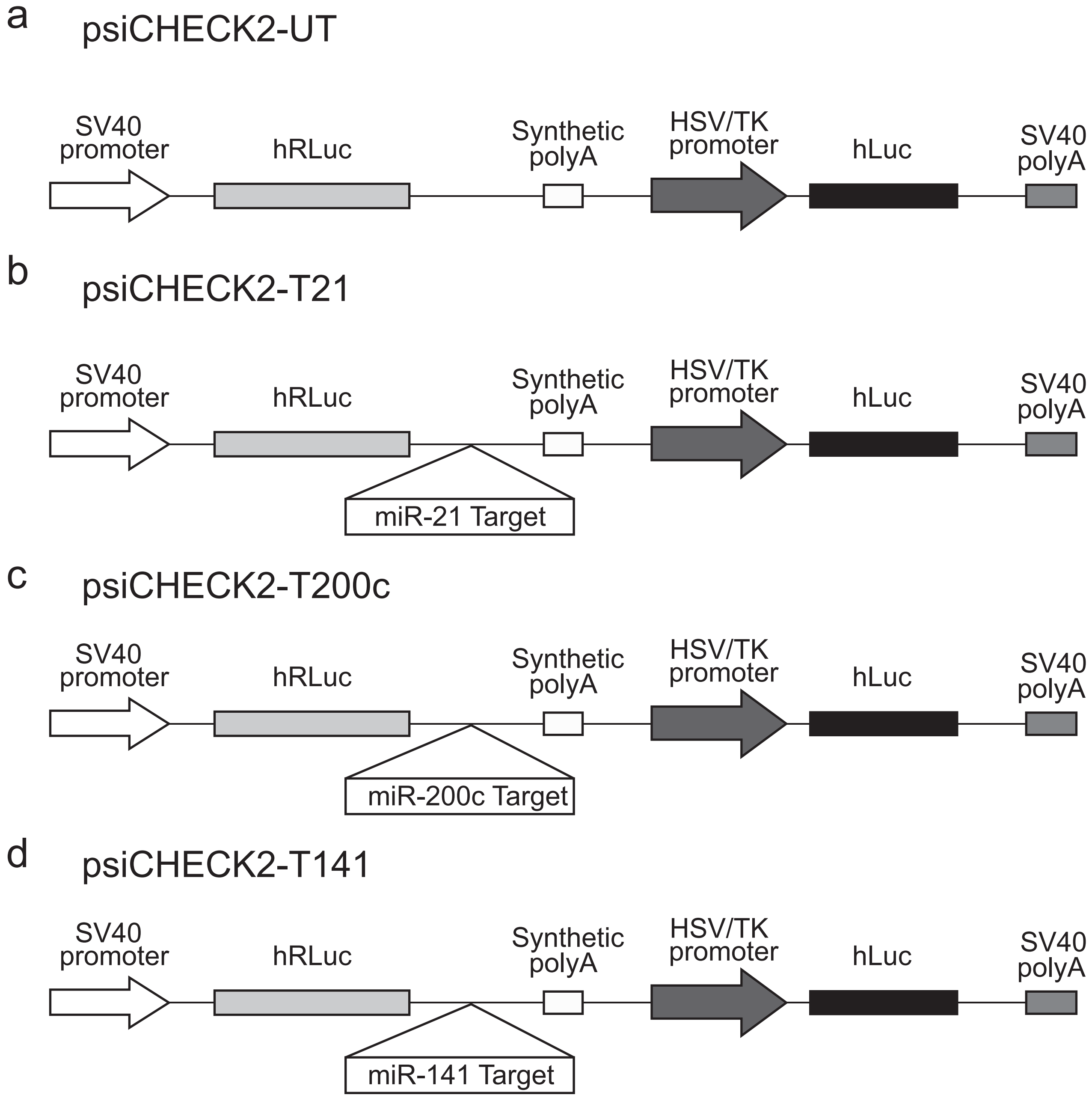

Supplementary Figure S3

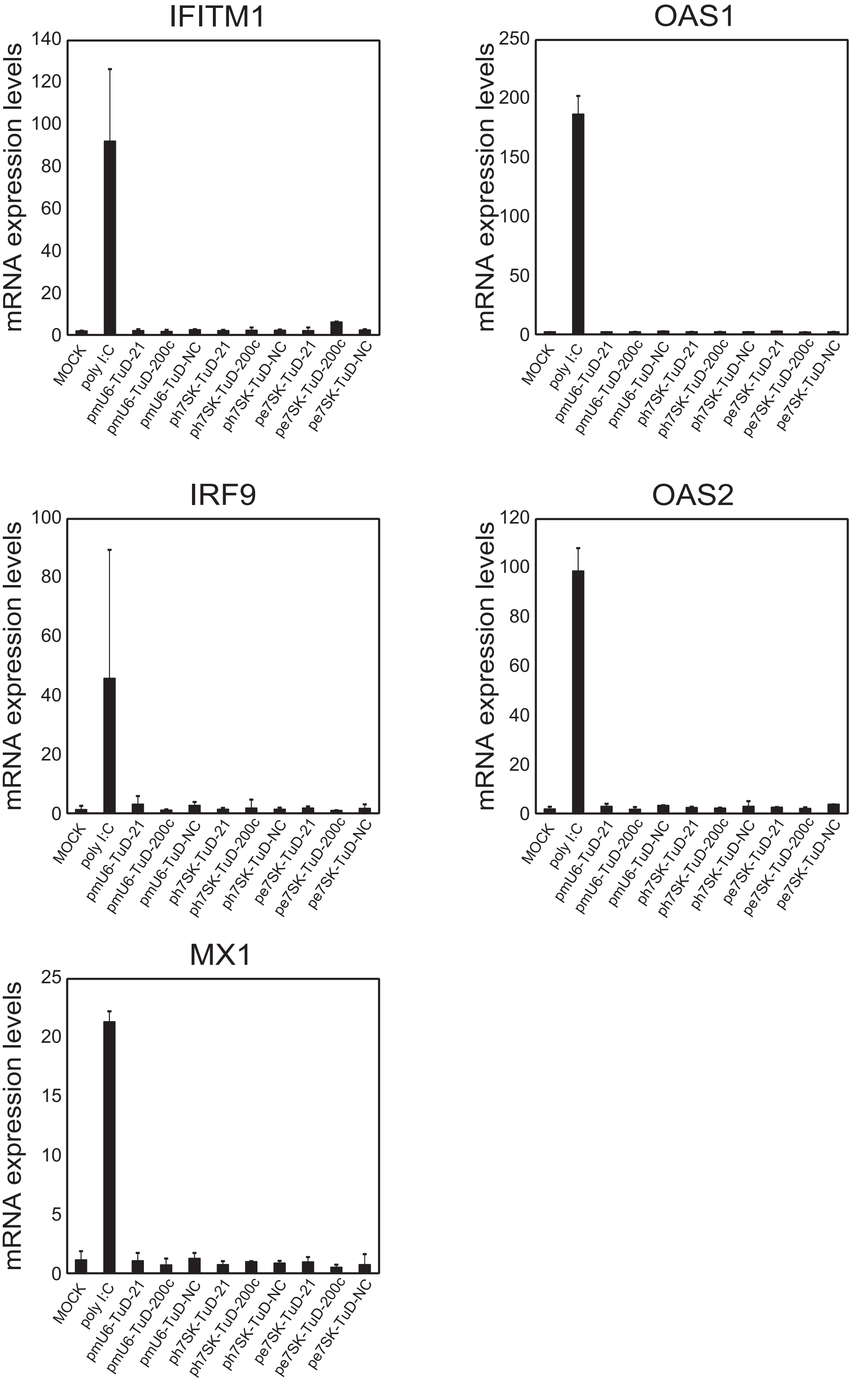

Supplementary Figure S4

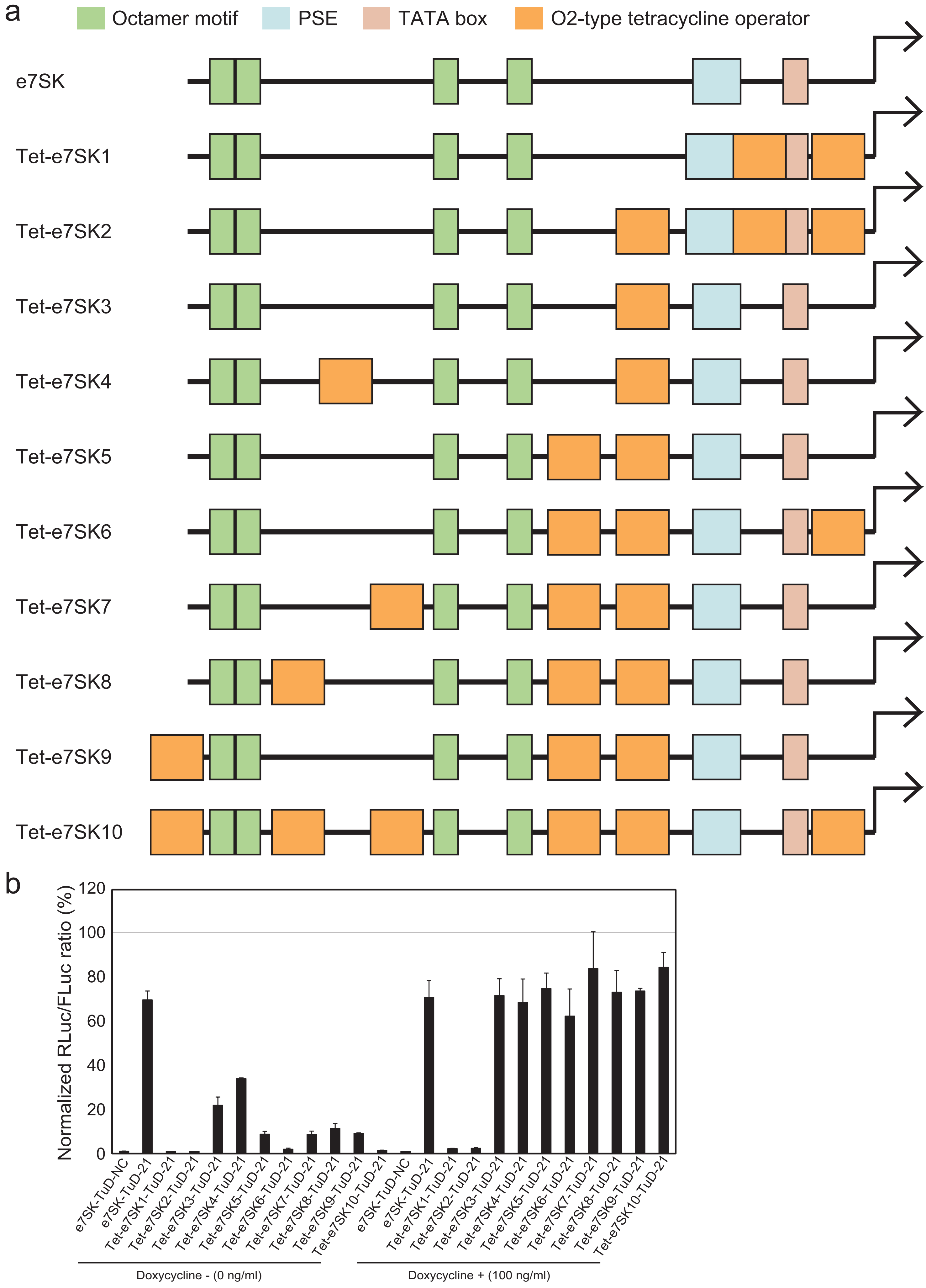

Supplementary Figure S5

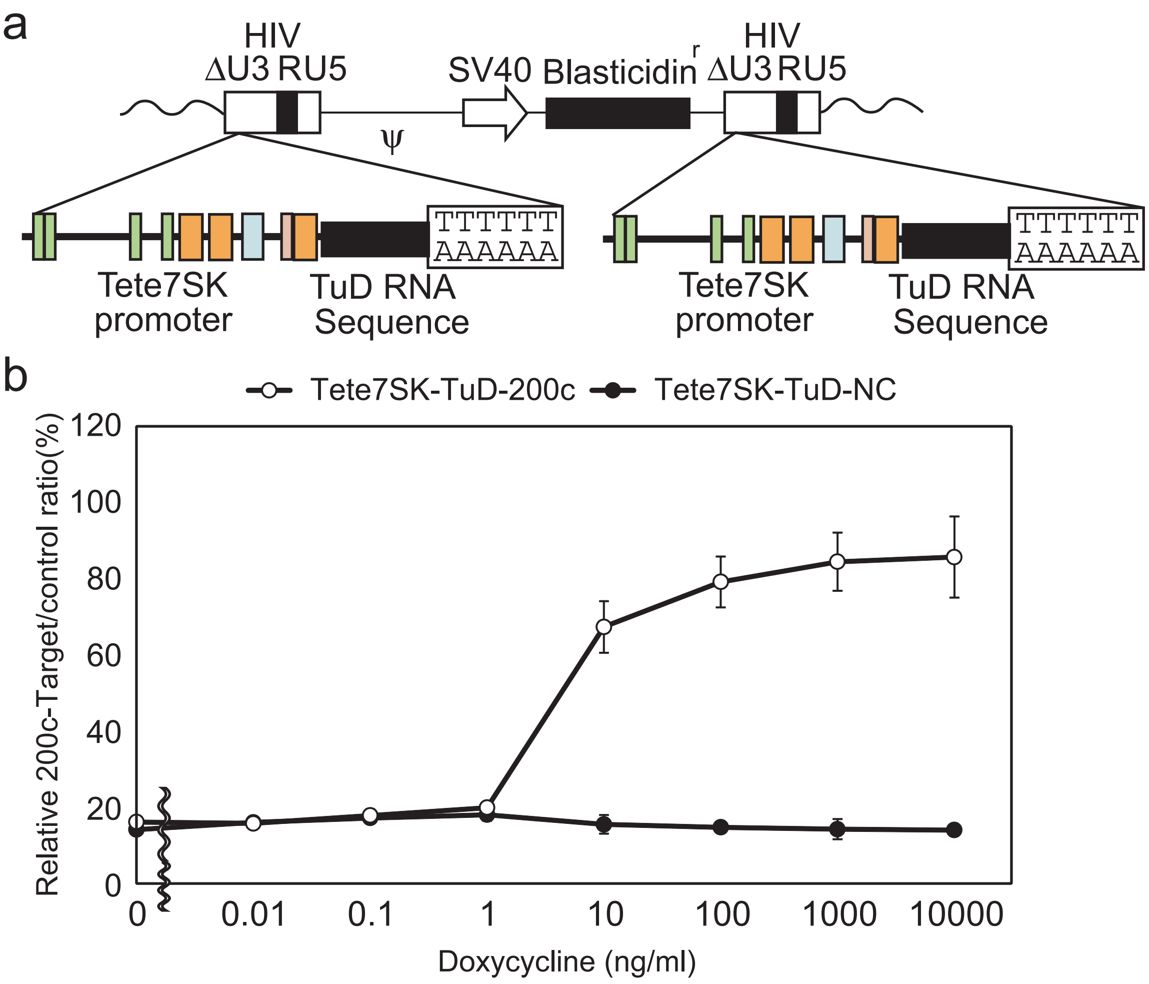

Supplementary Figure S6

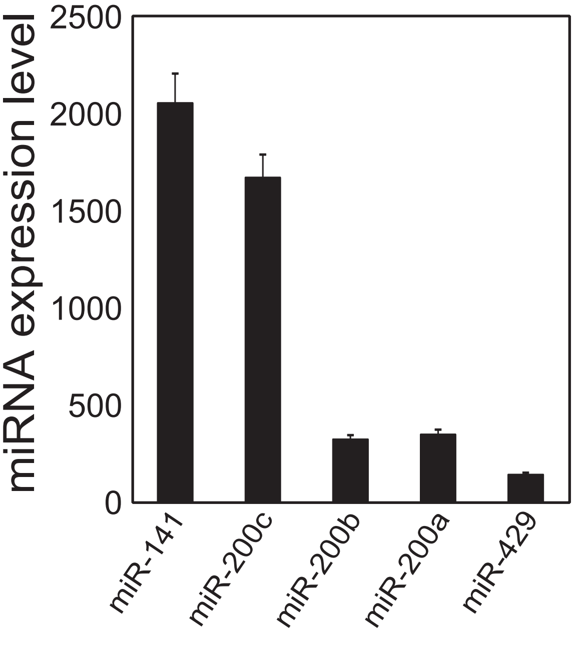

Supplementary Figure S7

TuD-141/200c TuD-NC

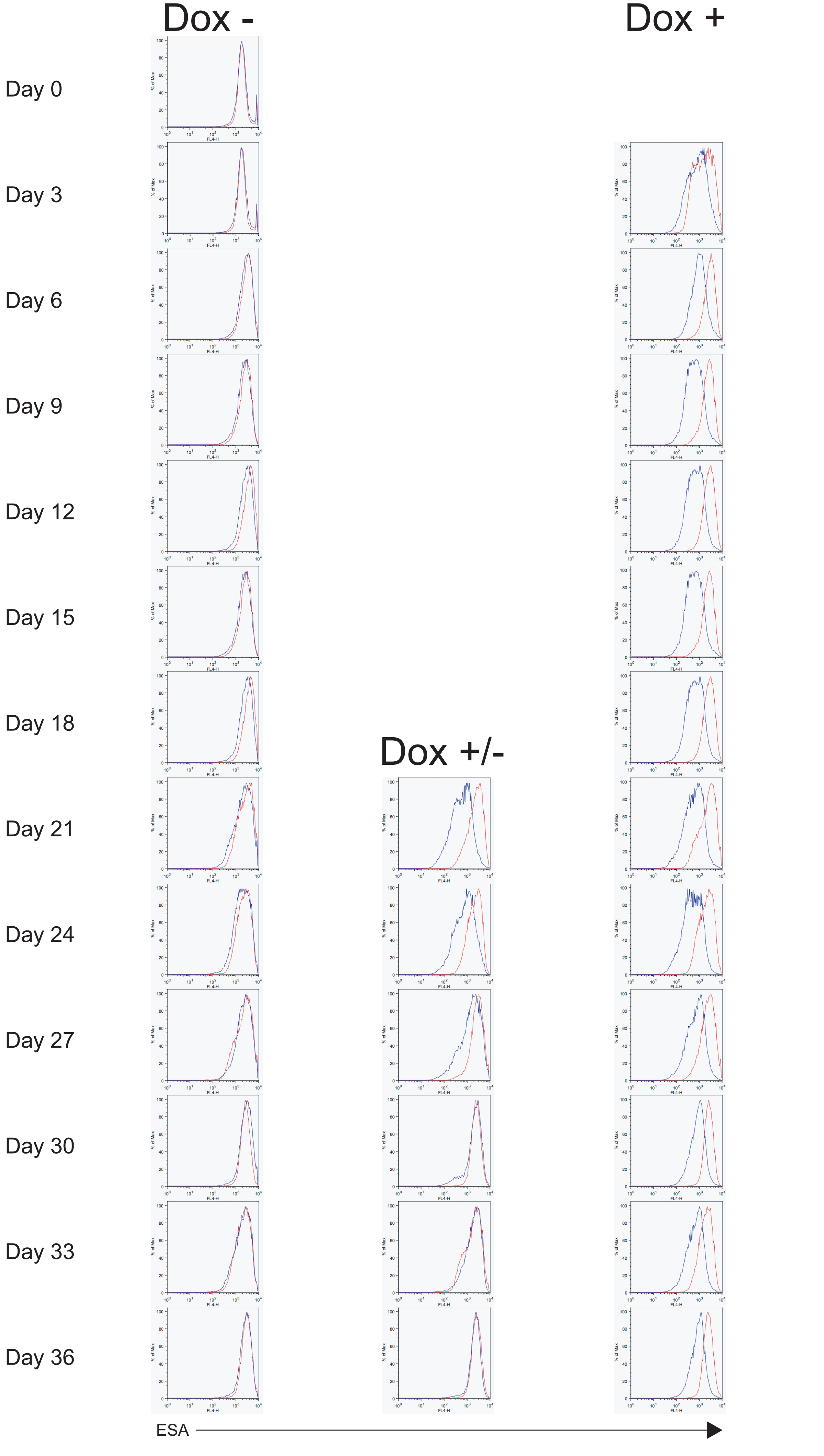

Supplementary Figure S8

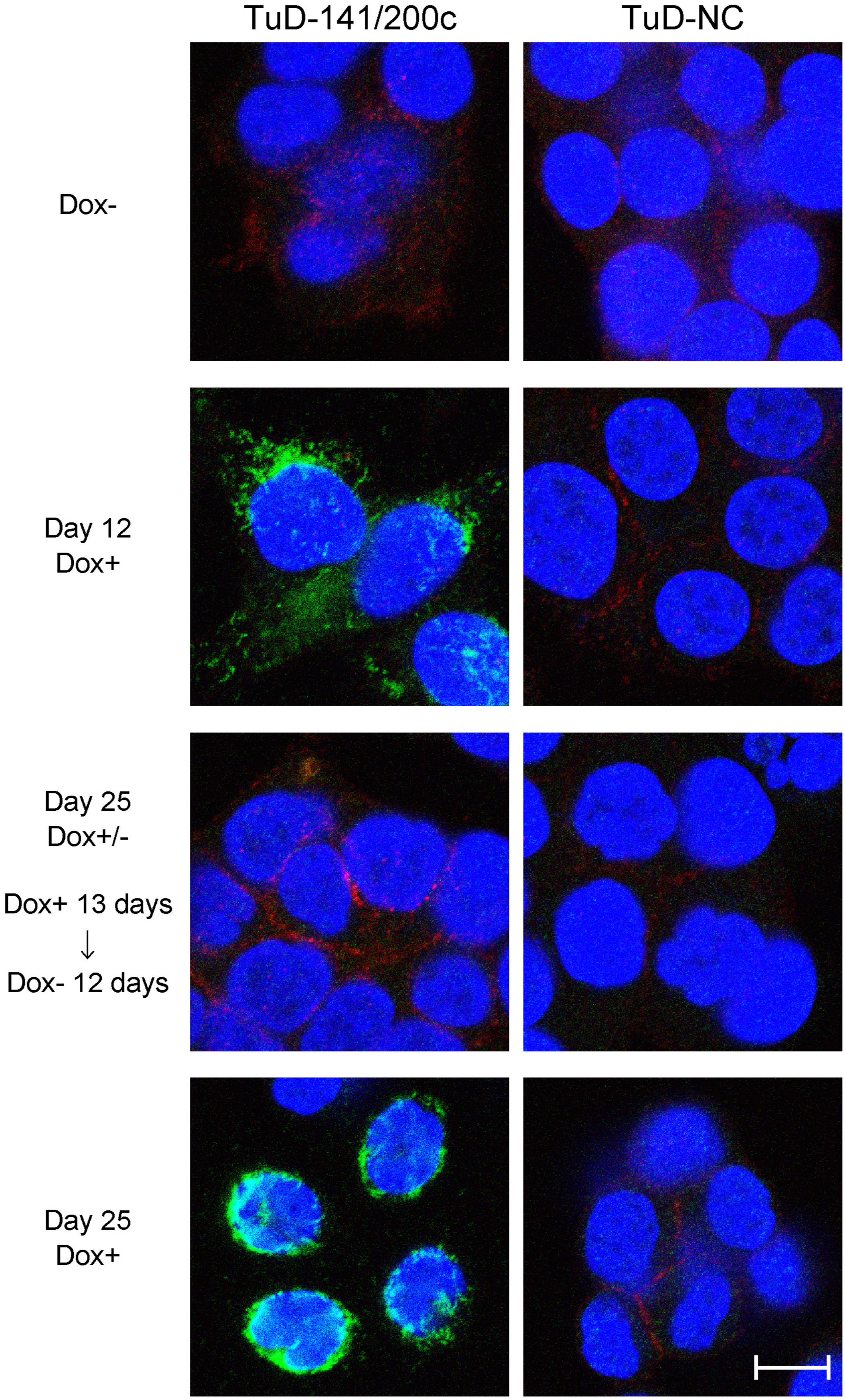

Supplementary Figure S9

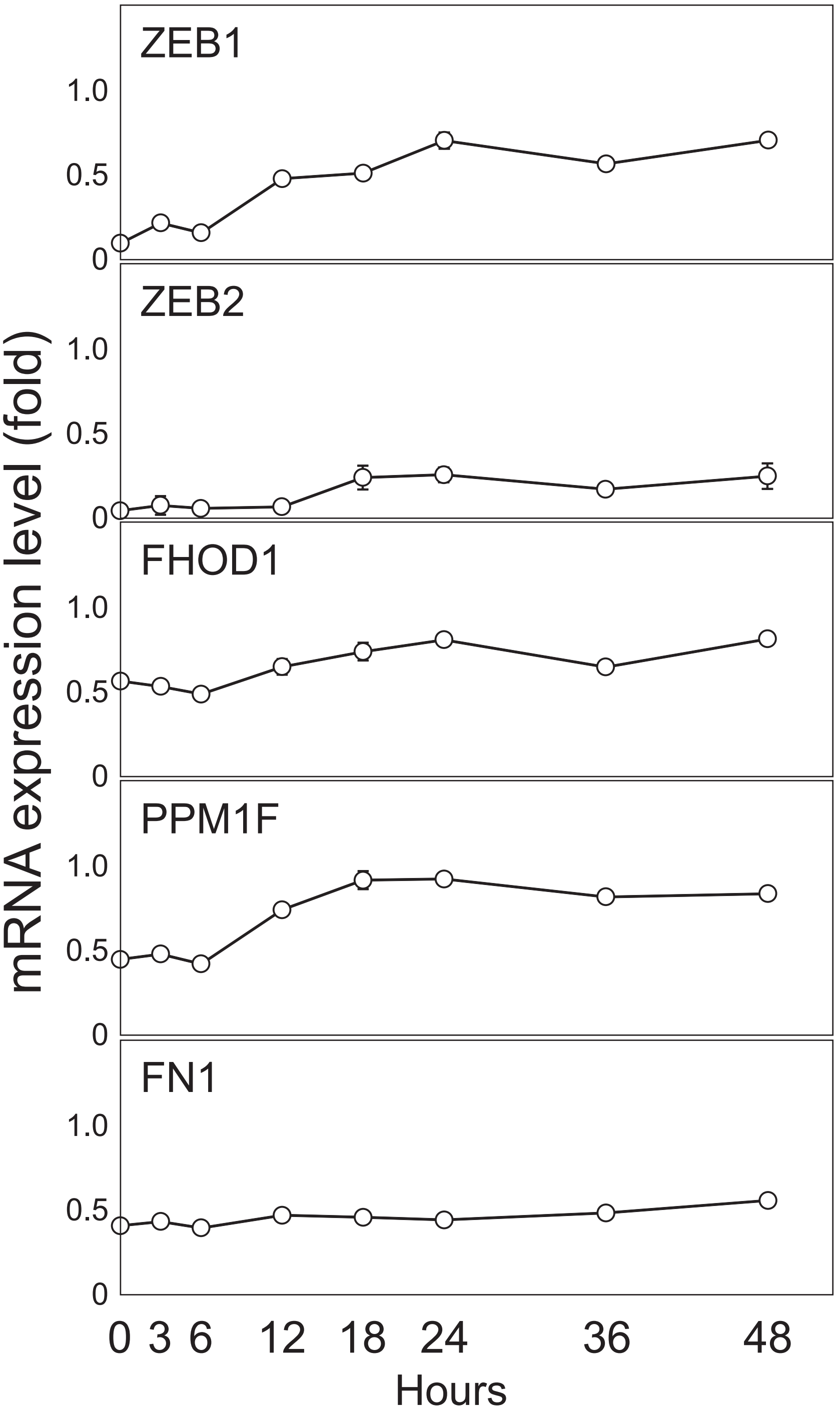

Supplementary Figure S10

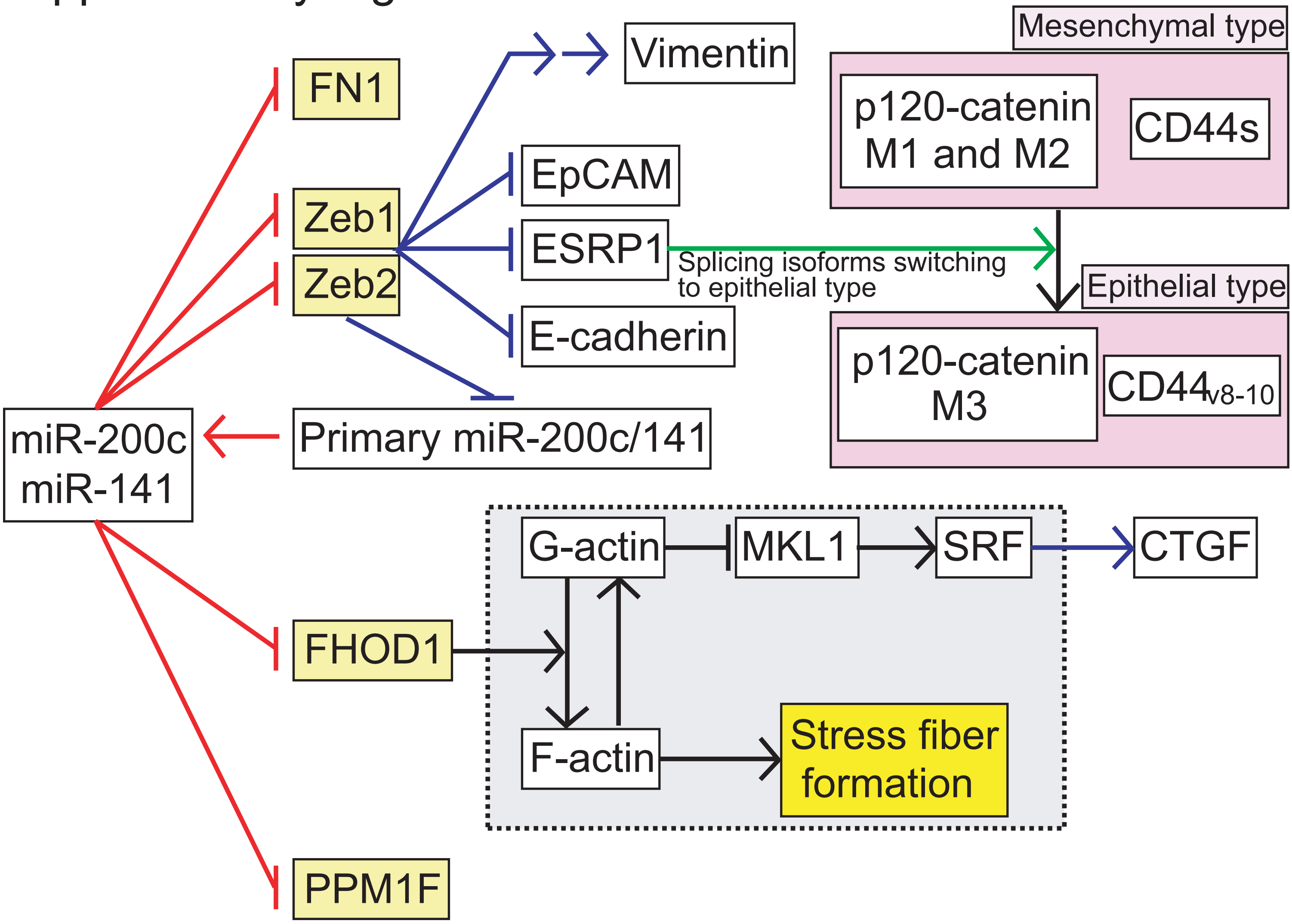

Supplementary Figure S11

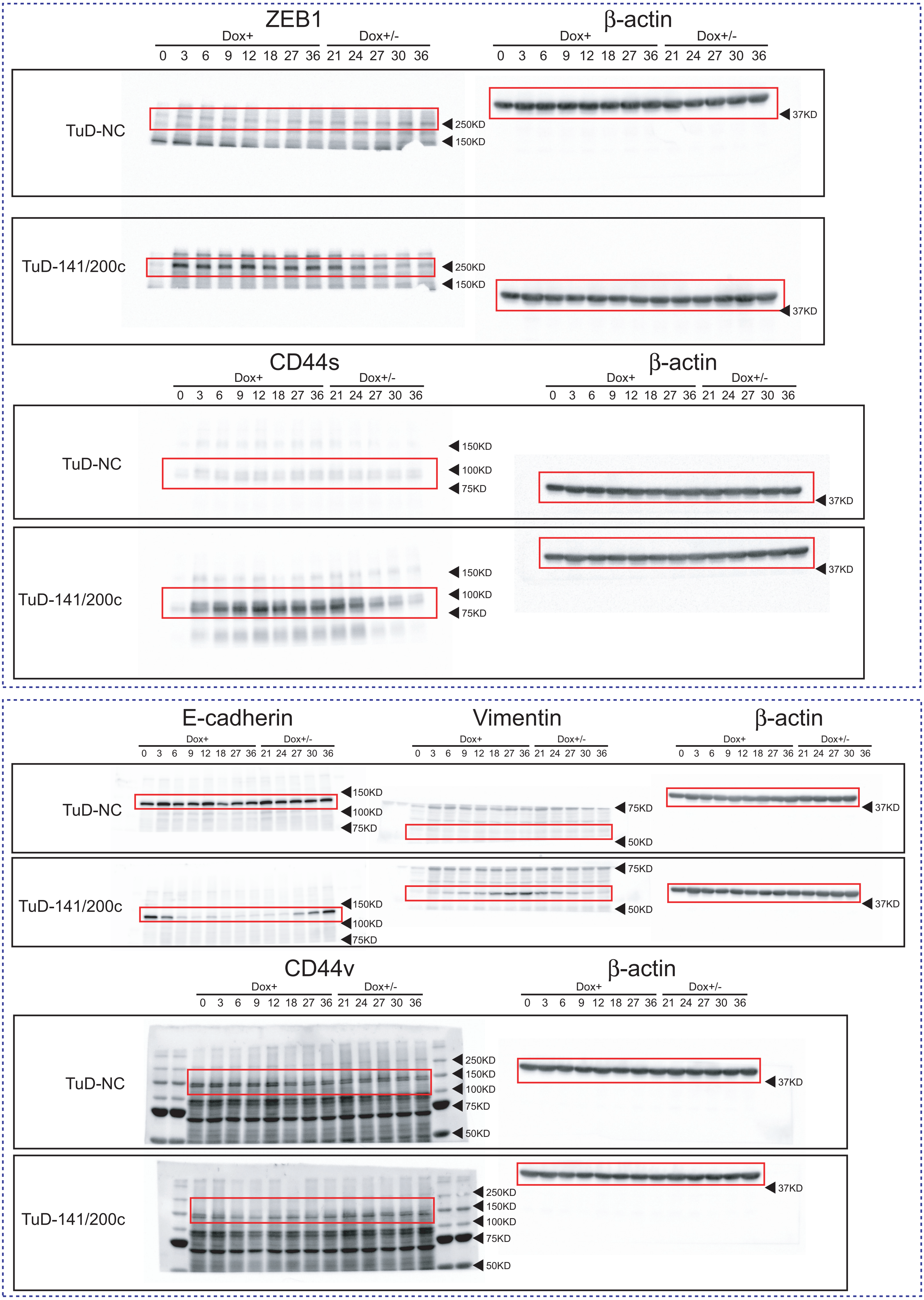

Supplementary Table S1. Synthesized DNA sequences for PolIII-driven TuD RNA expression vectors.

Primers		Sequence	
BamHI-H1-TuD-shuttle-EcoRI	5'–	GGATCCGAACGCTGACGTCATCAACCCGCTCCAAGGAATCGCGGGCCCAGTGTCACTAGGCGGGAACACCCAGC<br>GCGCGTGCGCCCTGGCAGGAAGATGGCTGTGAGGGACAGGGGAGTGGCGCCCTGCAATATTTGCATGTCGCTAT<br>GTGTTCTGGGAAATCACCATAAACGTGAAATGTCTTTGGATTTGGGAATCTTATAAGTTCTGTATGAGACCACAGG<br>ATGTGAGGGCGTCATCGGAGACGACACCATCCACAGCCAGCGTCTCGATGACGCCCTCACATCCTTTTTTGAA[illegible]	–3'
BamHI-e7SK-TuD-shuttle-EcoRI	5'–	GGATCCTGCAGTATTTGCATATGCAAATAAGGTGGTGGATCGATTCTGGATAGTGTCAAAACAGCCGGAAATCAA<br>GTCCGTTTATCTCAAACATTTGCATTTTGGGAATAAATGATATTTGCATTGCTGGTTAAATTAGATTTTAGTTAAATTT<br>CCTGCTGAAGCTCTAGTACGATAAGCAACTTGACCTAAGTGTAAAGTTGAGATTTCCTTCAGGTTTATATAGCTTGT<br>GCGCCGCCTGGGTACCTCGGATGTGAGGGCGTCATCGGAGACGACACCATCCACAGCCAGCGTCTCGATGACGCC<br>CTCACATCCTTTTTTGAATTC	–3'
BamHI-Tete7SK-TuD-shuttle-EcoRI	5'–	GGATCCTGCAGTATTTGCATATGCAAATAAGGTGGTGGATCGATTCTGGATAGTGTCAAAACAGCCGGAAATCAA<br>GTCCGTTTATCTCAAACATTTGCATTTTGGGAATAAATGATATTTGCATTGCTCCCTATCAGTGATAGAGATTAAATT<br>TCCTTCCCTATCAGTGATAGAGAAAGCAACTTGACCTAAGTGTAAAGTTGAGATTTCCTTCAGGTTTATATAGCTCC<br>CTATCAGTGATAGAGACTCGGATGTGAGGGCGTCATCGGAGACGACACCATCCACAGCCAGCGTCTCGATGACG<br>CCCTCACATCCTTTTTTGAATTC	–3'

Supplementary Table S2. Primer pairs used for TuD RNA expression vectors.

Primers		Sequence
TuD-21	s^+	5’- CATCAACTCAACATCAGTCAATGTGATAAGCTACAAGTATTCTGGTCACAGAATACAACTCAACATCAGTCAATGTGATAAGCTACAAG -3’
TuD-21	a^+	5’- TCATCTTGTAGCTTATCACATTGACTGATGTTGAGTTGTATTCTGTGACCAGAATACTTGTAGCTTATCACATTGACTGATGTTGAGTT -3’
TuD-200c	s	5’- CATCAACTCCATCATTACCCATTAGGCAGTATTACAAGTATTCTGGTCACAGAATACAACTCCATCATTACCCATTAGGCAGTATTACAAG -3’
TuD-200c	a	5’- TCATCTTGTAATACTGCCTAATGGGTAATGATGGAGTTGTATTCTGTGACCAGAATACTTGTAATACTGCCTAATGGGTAATGATGGAGTT -3’
TuD-141/200c	s	5’- CATCAACCCATCTTTACCACATAGACAGTGTTACAAGTATTCTGGTCACAGAATACAACTCCATCATTACCCCACTGGCAGTATTACAAG -3’
TuD-141/200c	a	5’- TCATCTTGTAATACTGCCAGTGGGGTAATGATGGAGTTGTATTCTGTGACCAGAATACTTGTAACACTGTCTATGTGGTAAAGATGGGTT -3’
TuD-NC	s	5’- CATCAACTATCGCGAGTATCGACGTCGAGGCCCAAGTATTCTGGTCACAGAATACAACTATCGCGAGTATCGACGTCGAGGCCCAAG -3’
TuD-NC	a	5’- TCATCTTGGGCCTCGACGTCGATACTCGCGATAGTTGTATTCTGTGACCAGAATACTTGGGCCTCGACGTCGATACTCGCGATAGTT -3’

s^+; sense strand

a^+; antisense strand

Supplementary Table S3. Primer pairs used for luciferase reporter vectors.

Primers		Sequence
psiCHECK2-T21	s+	5'- TCGAGTCAACATCAGTCTGATAAGCTAGC -3'
psiCHECK2-T21	a+	5'- GGCCGCTAGCTTATCAGACTGATGTTGAC -3'
psiCHECK2-T200c	s	5'- TCGAGTCCATCATTACCCGGCAGTATTAGC -3'
psiCHECK2-T200c	a	5'- GGCCGCTAATACTGCCGGGTAATGATGGAC -3'
psiCHECK2-T141	s	5'- TCGAGCCATCTTTACCAGACAGTGTTAGC -3'
psiCHECK2-T141	a	5'- GGCCGCTAACACTGTCTGGTAAAGATGGC -3'

s+; sense strand

a+; antisense strand

Supplementary Table S4. Primer pairs used for Real time PCR.

Primers		Sequence
OAS1	F+	5'-CACCAAGCTCAAGAGCCTCA-3'
OAS1	R+	5'-CCCAAGCATAGACCGTCAGG-3'
OAS2	F	5'-TACCACCTTGGAAAGTGCCG-3'
OAS2	R	5'-GGCTTCTGGATGAGAACATCT-3'
MX1	F	5'-TCCGGCTTGCTTTCACAGAT-3'
MX1	R	5'-GCACCCCTGTATACCTGGTC-3'
IRF9	F	5'-GGGAGCAGTCCATTCAGACA -3'
IRF9	R	5'-CAGCAGTGAGTAGTCTGGCT -3'
IFITM	F	5'-TCCGTGAAGTCTAGGGACAGG-3'
IFITM	R	5'-GAGCCGAATACCAGTAACAGGA -3'
Zeb1	F	5'-CACACCAGAAGCCAGTGGTC-3'
Zeb1	R	5'-AACTGCACAGGGAGCAACTA-3'
Zeb2	F	5'-ACATCAAGTACCGCCACGAG-3'
Zeb2	R	5'-GCATTTGGTGCTGATCTGTCC-3'
FHOD1	F	5'-TGAGTCTCGTGCCAAAGAGG-3'
FHOD1	R	5'-ACAGGTGGCAGTGTGGTTAG-3'
PPM1F	F	5'-GCATTGGGTGGCTTTGTGTC-3'
PPM1F	R	5'-CAGACACGTAGGGCTTCTGG-3'
FN1	F	5'-AGTGGAAGTGTGAGAGGCAC-3'
FN1	R	5'-TGAGGCTGCGGTTGGTAAAC-3'
Pri-miR-200c	F	5'-GCAGTAACCTTCAGGGAGCC-3'
Pri-miR-200c	R	5'-GATTCGTCCCCCATCCAGAG-3'
Vimentin	F	5'-GCTTCAGAGAGAGGAAGCCG-3'
Vimentin	R	5'-AAGGTCAAGACGTGCCAGAG-3'
E-cadherin	F	5'-ACGCCGAGAGCTACACGTTC-3'
E-cadherin	R	5'-TGAATCGGGTGTCGAGGGAA-3'
ESA	F	5'-GCTGGAATTGTTGTGCTGGTTA-3'
ESA	R	5'-AAGATGTCTTCGTCCCACGC-3'
ESRP1	F	5'-TGTAAGTGAGGAGCACCGAG-3'
ESRP1	R	5'-TGGAGAGAAACTGGGCTACC-3'
pan-CD44	F	5'-TGGCGCAGATCGATTTGAATA-3'
pan-CD44	R	5'-CCGTCCGAGAGATGCTGTAG-3'
CD44v8-10	F	5'-TCTTTCAATGACAACGCAGCA-3'
CD44v8-10	R	5'-TTGGGTCTCTTCTTCCACCTG-3'
CD44s	F	5'-TACACCCCATCCCAGACGAA-3'
CD44s	R	5'-GAATGTGTCTTGGTCTCTGGTAGC-3'
pan-p120	F	5'-TCCTGCCATCCTAGAAGCCT-3'
pan-p120	R	5'-GCAGAGCAGAGCGGATGTAT-3'
p120-M3	F	5'-CCGAAAGGAGGAAGAGGTGGCG-3'
p120-M3	R	5'-CAAACCGGCCGTTCTCAAATGTGG-3'
p120-M1/M2	F	5'-CTCGGCGCAGCTGGAACGCGTC-3'
p120-M1/M2	R	5'-CACAAACCGGCCGTTCTGATGC-3'
Snail	F	5'-GGACCCACACTGGCGAGAAG-3'
Snail	R	5'-ACATTCGGGAGAAGGTCCGA-3'
Slug	F	5'-CGAACTGGACACACATACAGTGA-3'
Slug	R	5'-GGAATGGAGCAGCGGTAGTC-3'
BMI1	F	5'-GACCAGAACAGATTGGATCGGA-3'
BMI1	R	5'-ATTGCTGCTGGGCATCGTAA-3'
Notch1	F	5'-GTGTATTGACGACGTTGCCG-3'
Notch1	R	5'-ATAGTCCTCGGATTGCCTGC-3'
CTGF	F	5'-GGAAGAGAACATTAAGAAGGGCA-3'
CTGF	R	5'-GTGCAGCCAGAAAGCTCAAA-3'
GAPDH	F	5'- ACTTTGTCAAGCTCATTTCCTG -3'
GAPDH	R	5'- CTCTCTTCCTCTTGTGCTCTTG -3'

F+; Forward primer

R+; Reverse primer

Supplementary Table S5. Synthesized DNA sequences for Tet-inducible e7SK-driven TuD RNA expression shuttle vectors.

Primers		Sequence	
BamHI-Tet-e7SK1-TuD-shuttle-EcoRI	5'−	GGATCCTGCAGTATTTGCATATGCAAATAAGGTGGTGGATCGATTCTGGATAGTGTCAAAACAGCCGGAAATCAA<br>GTCCGTTTATCTCAAACATTTGCATTTTGGGAATAAATGATATTTGCATTGCTGGTTAAATTAGATTTTAGTTAAATTT<br>CCTGCTGAAGCTCTAGTACGATAAGCAACTTGACCTAAGTGTAAAGTTGTCCCTATCAGTGATAGAGAGTTTATAT<br>AGCTCCCTATCAGTGATAGAGACTCGGATGTGAGGGCGTCATCGGAGACGACACCATCCACAGCCAGCGTCTCG<br>ATGACGCCCTCACATCCTTTTTTGAATTC	−3'
BamHI-Tet-e7SK2-TuD-shuttle-EcoRI	5'−	GGATCCTGCAGTATTTGCATATGCAAATAAGGTGGTGGATCGATTCTGGATAGTGTCAAAACAGCCGGAAATCAA<br>GTCCGTTTATCTCAAACATTTGCATTTTGGGAATAAATGATATTTGCATTGCTGGTTAAATTAGATTTTAGTTAAATTT<br>CCTTCCCTATCAGTGATAGAGAAAGCAACTTGACCTAAGTGTAAAGTTGTCCCTATCAGTGATAGAGAGTTTATATA<br>GCTCCCTATCAGTGATAGAGACTCGGATGTGAGGGCGTCATCGGAGACGACACCATCCACAGCCAGCGTCTCGAT<br>GACGCCCTCACATCCTTTTTTGAATTC	−3'

Supplementary Table S6. Synthesized DNA sequences for Tet-inducible e7SK-driven TuD-21 expression vectors.

Primers		Sequence
BamHI-Tet-e7SK3-TuD-21-EcoRI	5’–	GGATCCTGCAGTATTTGCATATGCAAATAAGGTGGTGGATCGATTCTGGATAGTGTCAAAACAGCCGGAAATCAA<br>GTCCGTTTATCTCAAACATTTGCATTTTGGGAATAAATGATATTTGCATTGCTGGTTAAATTAGATTTTAGTTAAATT<br>TCCTTCCCTATCAGTGATAGAGAAAGCAACTTGACCTAAGTGTAAAGTTGAGATTTCCTTCAGGTTTATATAGCTTG<br>TGCGCCGCCTGGGTACCTCGGATGTGAGGGCGTCATCAACTCAACATCAGTCAATGTGATAAGCTACAAGTATTC<br>TGGTCACAGAATACAACTCAACATCAGTCAATGTGATAAGCTACAAGATGACGCCCTCACATCCTTTTTTGAAT –3’
BamHI-Tet-e7SK4-TuD-21-EcoRI	5’–	GGATCCTGCAGTATTTGCATATGCAAATAAGGTGGTGGATCGATTCTGGATAGTGTCATCCCTATCAGTGATAGAG<br>ACCGTTTATCTCAAACATTTGCATTTTGGGAATAAATGATATTTGCATTGCTGGTTAAATTAGATTTTAGTTAAATTT<br>CCTTCCCTATCAGTGATAGAGAAAGCAACTTGACCTAAGTGTAAAGTTGAGATTTCCTTCAGGTTTATATAGCTTGT<br>GCGCCGCCTGGGTACCTCGGATGTGAGGGCGTCATCAACTCAACATCAGTCAATGTGATAAGCTACAAGTATTCT<br>GGTCACAGAATACAACTCAACATCAGTCAATGTGATAAGCTACAAGATGACGCCCTCACATCCTTTTTTGAATT –3’
BamHI-Tet-e7SK5-TuD-21-EcoRI	5’–	GGATCCTGCAGTATTTGCATATGCAAATAAGGTGGTGGATCGATTCTGGATAGTGTCAAAACAGCCGGAAATCAA<br>GTCCGTTTATCTCAAACATTTGCATTTTGGGAATAAATGATATTTGCATTGCTCCCTATCAGTGATAGAGATTAAATT<br>TCCTTCCCTATCAGTGATAGAGAAAGCAACTTGACCTAAGTGTAAAGTTGAGATTTCCTTCAGGTTTATATAGCTTG<br>TGCGCCGCCTGGGTACCTCGGATGTGAGGGCGTCATCAACTCAACATCAGTCAATGTGATAAGCTACAAGTATTCT<br>GGTCACAGAATACAACTCAACATCAGTCAATGTGATAAGCTACAAGATGACGCCCTCACATCCTTTTTTGAATT –3’
BamHI-Tet-e7SK6-TuD-21-EcoRI	5’–	GGATCCTGCAGTATTTGCATATGCAAATAAGGTGGTGGATCGATTCTGGATAGTGTCAAAACAGCCGGAAATCAA<br>GTCCGTTTATCTCAAACATTTGCATTTTGGGAATAAATGATATTTGCATTGCTCCCTATCAGTGATAGAGATTAAATT<br>TCCTTCCCTATCAGTGATAGAGAAAGCAACTTGACCTAAGTGTAAAGTTGAGATTTCCTTCAGGTTTATATAGCTCC<br>CTATCAGTGATAGAGACTCGGATGTGAGGGCGTCATCAACTCAACATCAGTCAATGTGATAAGCTACAAGTATTC<br>TGGTCACAGAATACAACTCAACATCAGTCAATGTGATAAGCTACAAGATGACGCCCTCACATCCTTTTTTGAAT –3’
BamHI-Tet-e7SK7-TuD-21-EcoRI	5’–	GGATCCTGCAGTATTTGCATATGCAAATAAGGTGGTGGATCGATTCTGGATAGTGTCAAAACAGCCGGAAATTCC<br>CTATCAGTGATAGAGACATTTGCATTTTGGGAATAAATGATATTTGCATTGCTCCCTATCAGTGATAGAGATTAAA<br>TTTCCTTCCCTATCAGTGATAGAGAAAGCAACTTGACCTAAGTGTAAAGTTGAGATTTCCTTCAGGTTTATATAGCT<br>TGTGCGCCGCCTGGGTACCTCGGATGTGAGGGCGTCATCAACTCAACATCAGTCAATGTGATAAGCTACAAGTAT<br>TCTGGTCACAGAATACAACTCAACATCAGTCAATGTGATAAGCTACAAGATGACGCCCTCACATCCTTTTTTGAAT<br>TC –3’
BamHI-Tet-e7SK8-TuD-21-EcoRI	5’–	GGATCCTGCAGTATTTGCATATGCAAATAAGGTGGTGGATCGATCCCTATCAGTGATAGAGAAGCCGGAAATCAA<br>GTCCGTTTATCTCAAACATTTGCATTTTGGGAATAAATGATATTTGCATTGCTCCCTATCAGTGATAGAGATTAAATT<br>TCCTTCCCTATCAGTGATAGAGAAAGCAACTTGACCTAAGTGTAAAGTTGAGATTTCCTTCAGGTTTATATAGCTTG<br>TGCGCCGCCTGGGTACCTCGGATGTGAGGGCGTCATCAACTCAACATCAGTCAATGTGATAAGCTACAAGTATTCT<br>GGTCACAGAATACAACTCAACATCAGTCAATGTGATAAGCTACAAGATGACGCCCTCACATCCTTTTTTGAATT –3’
BamHI-Tet-e7SK9-TuD-21-EcoRI	5’–	GGATCCTCCCTATCAGTGATAGAGAGTATTTGCATATGCAAATAAGGTGGTGGATCGATTCTGGATAGTGTCAAAA<br>CAGCCGGAAATCAAGTCCGTTTATCTCAAACATTTGCATTTTGGGAATAAATGATATTTGCATTGCTCCCTATCAGTG<br>ATAGAGATTAAATTTCCTTCCCTATCAGTGATAGAGAAAGCAACTTGACCTAAGTGTAAAGTTGAGATTTCCTTCAG<br>GTTTATATAGCTTGTGCGCCGCCTGGGTACCTCGGATGTGAGGGCGTCATCAACTCAACATCAGTCAATGTGATAA<br>GCTACAAGTATTCTGGTCACAGAATACAACTCAACATCAGTCAATGTGATAAGCTACAAGATGACGCCCTCACAT<br>CCTTTTTTGAATTC –3’
BamHI-Tet-e7SK10-TuD-21-EcoRI	5’–	GGATCCTCCCTATCAGTGATAGAGAGTATTTGCATATGCAAATAAGGTGGTGGATCGATCCCTATCAGTGATAGAG<br>AAGCCGGAAATTCCCTATCAGTGATAGAGACATTTGCATTTTGGGAATAAATGATATTTGCATTGCTCCCTATCAGT<br>GATAGAGATTAAATTTCCTTCCCTATCAGTGATAGAGAAAGCAACTTGACCTAAGTGTAAAGTTGAGATTTCCTTCA<br>GGTTTATATAGCTCCCTATCAGTGATAGAGACTCGGATGTGAGGGCGTCATCAACTCAACATCAGTCAATGTGATA<br>AGCTACAAGTATTCTGGTCACAGAATACAACTCAACATCAGTCAATGTGATAAGCTACAAGATGACGCCCTCACA<br>TCCTTTTTTGAATTC –3’
